# Increasing digital media visibility and tourism messaging promote US National Park system integration

**DOI:** 10.1093/pnasnexus/pgag028

**Published:** 2026-02-10

**Authors:** Alexander Michael Petersen, Felber Arroyave, Stephen Shackelton, Jeffrey Scott Jenkins

**Affiliations:** Department of Management of Complex Systems, Ernest and Julio Gallo Management Program, School of Engineering, University of California Merced, 5200 North Lake Rd., Merced, CA 95343, USA; National Parks Institute, University of California Merced, 5200 North Lake Rd., Merced, CA 95343, USA; Department of Management of Complex Systems, Ernest and Julio Gallo Management Program, School of Engineering, University of California Merced, 5200 North Lake Rd., Merced, CA 95343, USA; National Parks Institute, University of California Merced, 5200 North Lake Rd., Merced, CA 95343, USA; Department of Management of Complex Systems, Ernest and Julio Gallo Management Program, School of Engineering, University of California Merced, 5200 North Lake Rd., Merced, CA 95343, USA; National Parks Institute, University of California Merced, 5200 North Lake Rd., Merced, CA 95343, USA

**Keywords:** protected areas, systems science, sustainability management, computational social science

## Abstract

The National Park Service (NPS) faces a paradoxical dual mandate—to preserve invaluable environmental and cultural resources for future generations and to ensure their public accessibility for recreational enjoyment. Yet with >124 million visitors in 2019, the US national parks (NPs) are at risk of being “loved to death” a challenge faced by protected areas the world over. This growing demand for ecosystem services calls for new strategies to enhance public appreciation and commitment to protecting natural capital. Against this backdrop, we analyzed the structure and dynamics of the NP system through a public-facing lens constructed from >426,000 digital media articles mentioning at least one park by its official name. Our analysis reveals that from 2010 to 2019, NP media visibility grew by over 3,900%, outpacing 29% growth in visitation, and a 15% decline in federal budget support for NPs. We find that this disproportionate media growth is driven by tourism-oriented articles referencing multiple NPs, which has become the principal driver of NP system integration in the public sphere. Consequently, ecotourism marketing has displaced public attention from critical issues associated with environmental, wildfire and wildlife management. With many NPs operating at or near capacity, tourism-driven integration of the NP system may intensify fundamental tensions within the NPS dual mandate, as rising demand for ecosystem services collides with limited federal resources and growing environmental risks.

Significance Statement We exploit a comprehensive set of 426,000 digital media articles referencing at least one of 63 US national parks (NPs) by name to construct a dynamic representation of this protected area system, which thereby facilitates understanding the conservation–recreation paradox from a public perspective. From 2010 to 2019, media visibility increased by more than 3,900%, with tourism-oriented articles becoming the principal driver of NP system integration in the public sphere. Consequently, ecotourism marketing has displaced public attention from critical issues associated with environmental, wildfire, and wildlife management. As tourism narratives increasingly shape public engagement with national parks, rising visitation pressures call for increased federal support to bolster the National Park Service’s capacity to manage resources and conserve the nation’s shared natural heritage.

## Introduction

National parks (NPs) are naturally replenishing sources of wonderment and outdoors experience that foster rich emotional and educational experiences that are highly personalized and readily shared (1). By prioritizing public accessibility and outdoor recreation alongside environmental conservation, NPs are fundamental to shaping public perception, identity, and imagination of wildlands and cultural landscapes as a shared national heritage (2–4). The considerable use and preservation values generated by NPs are tied to natural and cultural resources, and while concessionaire and entrance fees generate income from tourism demand, these metrics fail to account for the total value of ecosystem services, thereby underfunding investment in natural capital (5–8). However, the coupling of socioeconomic and environmental protection objectives introduces paradoxical tensions that confronts NP management with a challenging balancing act (5, 9–11). In the United States, this paradox is formalized in the 1916 Organic Act that charges the National Park Service (NPS) with its dual mandate “to conserve the scenery and the natural and historic objects and the wild life therein and to provide for the enjoyment of the same in such manner and by such means as will leave them unimpaired for the enjoyment of future generations” (4, 12).

In this regard, NP systems operate at the intersection of two objectives—balancing conservation goals with the demands of sustainable tourism, while also navigating the tension between park-specific priorities and broader system-wide policy (13). Although the NPS has discretionary authority to carry out its mission within park boundaries—including prioritizing the conservation mandate over public use in its management policies and related legal conflicts (14)—the cultural, societal, and economic value generated by NPs extends well beyond these physical limits (5, 6). This broader halo effect attracts tourism industry expansion at park peripheries, and contributes to the blurring of ecological, governance, and crisis management boundaries (15–19). At the same time, NPS governance must account for significant variation in biogeographical characteristics of individual parks—such as size, shape, and ecological diversity—while also addressing disparities in personnel and other critical management resources (20–22). These factors collectively inform federal budget requests and internal policy guidance, shaping how parks function both individually and as part of a larger system. Given these complex interdependencies, it is essential to understand the tourism and environmental management challenges facing NPs through a coupled human-environmental system lens that considers both individual park characteristics and broader system-wide dynamics.

To this end, we constructed a granular yet comprehensive representation of the US NP system by collecting >426,000 digital media articles published over the 21-year period 2000–2020 that each mention at least one of the 63 US NPs. This approach supports the application of topic modeling to analyze different parks through a consistent set of narratives, which thereby facilitates identifying how particular topics—in particular tourism vis-a-vis environmental messaging—enhances our understanding of the challenges facing NP system management according to its dual mandate.

While NPs are traditionally associated with their geographic embedding (23) anchored to place-based natural, historical, and cultural values, a principal advantage of our approach is to identify latent park–park relationships that extend across a wider range of political (environmental protection, governance), social (tourism, recreation), environmental (weather, wildlife) and infotainment (educational, entertainment) contexts. Our results reveal how the rise of tourism-focused media messaging drives system-level integration by linking individual parks through shared narratives and reinforcing their collective identity. Consequently, as media coverage increasingly emphasizes themes of natural beauty, recreation, and adventure, this framing is likely to (re)shape how public imaginaries form around principles and purpose of governance, conservation and visitation for parks and protected areas (PAs) the world over (24). By capturing these dynamics, our framework clarifies the role of sustainable tourism marketing in structuring and strengthening park system integration. This, in turn, can deepen public appreciation for NPs as a coupled human-environmental system, highlighting the interconnected challenges and opportunities associated with sustainability management.

## Materials and methods

### Descriptive statistics: composition of the US NP system

**Fig. 1.**
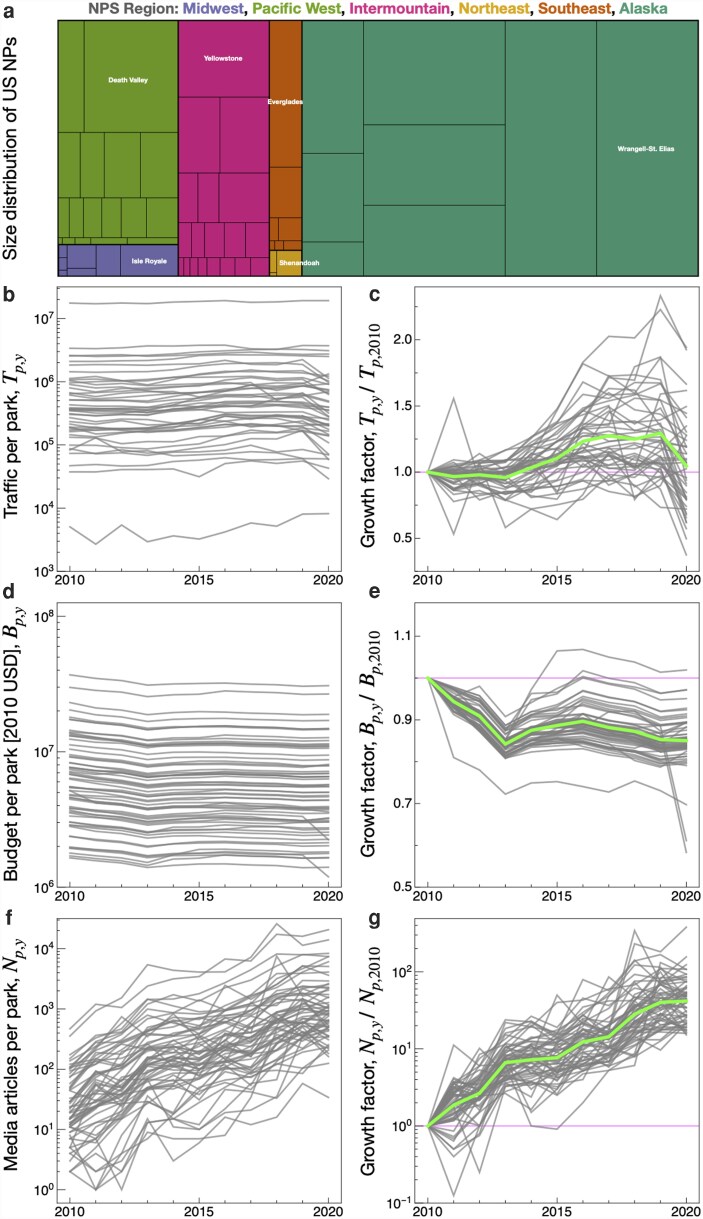
Trends in visitation traffic, federal budget and media visibility of US NPs. a) Size distribution of the gross area of 63 US NPs. b) The total vehicle traffic by year, based upon official records from the NPS which varies across nearly 4 orders of magnitude and exhibits the wide range of visitation levels conditioning NPS budget allocation and park management priorities (13). c) Growth of vehicle traffic, relative to 2010 levels. The thick curve represents the annual average: in 2019 the average increase in traffic was 29% above 2010 levels, ${\bar{T}}_{2019}/{\bar{T}}_{2010}=1.29$; however, in 2020 traffic levels fell back nearly to 2010 levels due to COVID-19 travel reduction combined with visitation permit systems implemented by the NPS, ${\bar{T}}_{2020}/{\bar{T}}_{2010}=1.05$. d) The US federal budget allocated to each park, $B_{\;p,y}$, deflated to constant 2010 US$. e) Park budget relative to 2010 levels, $B_{\;p,y}/B_{\;p,2010}$. Only one NP received a greater budget in 2020 relative to 2010, with the average NP receiving just 85% of its 2010 budget in both 2019 and 2020, ${\bar{B}}_{2019}/{\bar{B}}_{2010}\approx {\bar{B}}_{2020,y}/{\bar{B}}_{\;p,2010}=0.85$. f) The number of media articles in the MC database featuring a given park, $N_{\;p,y}$. g) Growth of park media visibility measured relative to 2010 levels, $N_{\;p,y}/N_{\;p,2010}$. On average, NP received over 3,900% more media visibility relative to 2010, ${\bar{N}}_{2019}/{\bar{N}}_{2010}=40.2$. See Fig. S5 for per-visitor trends, which are not substantially different.

The US NPs consists of 63 distinct administrative units characterized by an extremely broad size and geographic distribution—see Fig. 1a. Notably, the US NPs include landscapes that range from compact urban landmarks to vast wilderness preserves. These units are distributed not only across the continental US but also extend into US territories, including island and oceanic environments in the Pacific and Caribbean, thereby representing a geographically and ecologically diverse portfolio of protected areas. These 63 parks are classified as IUCN Category II protected areas, and together span ∼212,000 km^2^ in total (2% of US land area). As such, they make substantial contributions to the Aichi Biodiversity Target and 30×30 goals to expand global protected area coverage (25–27)—see Supplementary Information (SI) Fig. S1. The US NP system continues to expand, most recently with the addition of New River Gorge NP in 2020; and most prominently with the addition of Wrangell-St. Elias NP in 1980, which at (184 km)^2^ represents 16% of the total NP system area, and is roughly the size of Massachusetts.

### Data collection

In order to construct a granular yet comprehensive representation of US NP system from a public perspective, we used the Media Cloud project (MC) database (28), which provides a comprehensive representation of digital media. This open database is continuously constructed by crawling the internet, reaching a size of >1.7 billion searchable items in 2021 with a rate of roughly 1 million stories per day (29). Studies using MC data have examined both the relationship between media sources and their messaging on specific topics and events (30, 31), as well as the organization and evolution of media ecosystems composed of distinct identifiable entities (23, 32–34). To this end, we compiled a dataset of 426,069 unique web-based digital media articles produced by 16,030 unique media sources over the period 2000 to 2020, where each article mentions at least one US NP by its official name.

While the MC dataset is biased towards English-language content, because we are analyzing official US government entities, it is unlikely that this source language bias affects our representation of the US NP system. Of note, media articles collected by MC do not include social media content, which differ in terms of the content production sources, dissemination modes, multiplicity of distinct parks being featured in a single post, dynamic trend timescales, and consumer base. As such, MC data are not subject to biases associated with platform popularity, user demographics, willingness to make content publicly available, content brevity, and the social dynamics that drive content generation and consumption that are tangential to the NP context.

We collected metadata for a particular NP as follows. Starting with the official NP name, e.g. “Canyonlands National Park” or “Black Canyon of the Gunnison National Park,” we queried the MC API using Apache Solr syntax to identify articles featuring at least one exact text match in the full article text. As such, this name matching method depends on the consistent use of official NP names by professional writers, which is generally the case, and generates robust name recognition for the individual parks. Our sampling method does accommodate slight wording variations to increase the total recall associated with a given NP. For example, an article mentioning “Sequoia and Kings Canyon National Park” is appropriately identified for both parks since the Solr text query *“Sequoia National Park”∼7* (respectively, *“Kings Canyon National Park”∼7*) identifies matches within 7 tokens of each other.

### Article-level metadata for analyzing digital media visibility

While MC does not provide full article text due to copyright considerations, their API does supply the following metadata for each MC article (denoted by the index *a*):

1. the article title, $T_{a}$;
2. the weblink, ${\mathrm{URL}}_{a}$;
3. the publication date (defining the year $y_{a}$ and month $m_{a}$);
4. the media source $s_{a}$ publishing the article (e.g. New York Times, Washington Post);
5. article-level keywords ($e_{a}$) useful for identifying specific entities (“National Park Service,” “Senate”) and themes (e.g. “Environment,” “Accidents and Safety”).

Note that the entity keywords are not uniformly annotated, with only 14.5% of articles in our sample having at least one.

Together, these metadata enable the consistent measurement of each park’s digital media visibility, providing a contextually detailed proxy for brand equity that is derived from the natural, cultural, and historical features in the content of each article that are attributed to the park (32, 35–37). Since these media articles are sourced from ∼16,000 distinct media source providers, they are statistically representative of the broad set of contexts that characterize coupled human-environmental systems. Moreover, since each article has a distinct publication date, one can systematically track the evolution of media visibility ecosystem over time (32, 33).

On the aggregate, the NP system features a remarkable 3,900% growth in digital media visibility over the decade 2010–2019—see Fig. 1f and g. At the level of individual parks, the time series $N_{\;p,y}$ of media articles mentioning park *p* in year *y*, along with the aggregate total $N_{\;p}=\sum_{y}N_{\;p,y}$, together provide granular and consistent measures of digital media visibility. Figure S2a shows the temporal distribution of $N_{\;p,y}$ for each park, which collectively exhibit similar temporal trends as for the aggregated dataset shown in Fig. S3a and demonstrates the consistency of our sampling approach.

### Quantifying digital media co-visibility

To measure the pairwise relationship between any two parks, denoted by indices *i* and *j*, we first count the number of co-occurring media articles in a given period *y*, denoted by $N_{ij,y}$. The aggregated co-visibility is given by $N_{ij}=\sum_{y}N_{ij,y}$. By way of example, the maximum co-visibility observed across our entire sample is between Yellowstone and Grand Teton NPs, which are featured in $N_{ij}=$ 5,826 media articles together—see Fig. S2b. As in related studies of co-occurrence networks (32, 38, 39), we then quantify park–park co-visibility by way of the Jaccard similarity index,


(1) $$J_{ij,y}=\frac{N_{ij,y}}{N_{i,y}+N_{\;j,y}-N_{ij,y}}\in [0,1]\;,$$

which simply measures $N_{ij,y}$ as a fraction of the union of the two park’s article samples. Importantly, $J_{ij}$ accounts for both the wide variation in prominence across parks and the persistent secular growth of digital media content production. As such, $J_{ij}$ is bounded and intensive, which facilitates comparing the relative rate of co-visibility across parks and across time.

This method for quantifying park–park relationships facilitates analyzing both the dyadic and systems-level properties across the entire ensemble of NP relationships, which together form a dynamic weighted network (40). Considered individually, each $J_{ij}$ value measures the likelihood that a digital media consumer encountering an article about one or another park will find them both mentioned. By way of example, the co-visibility of Yellowstone and Grand Teton NPs is $J_{ij}=0.064$, meaning that they are co-mentioned in 100$J_{ij}=6.4$% of their merged article set. Considered together, the ensemble of all $J_{ij}$ values reveal the latent structure of the NP system (23), which extends well beyond the six formal NPS administrative regions (Northeast, Pacific West, Intermountain, Midwest, Southeast, and Alaska) that define NP groups according to geographic proximity.

### Topic classification of article titles

We use the combination of article-level keywords ($e_{a}$) and title ($T_{a}$) to ascertain media narratives communicated in each article. In order to generate a consistent representation of the NP topic space, we applied a natural language processing classifier to each $T_{a}$. The topic classifier is available in *Mathematica* software, and was trained on a corpus of Facebook posts, which are of similar length and contextual richness as article titles and other text sources commonly used in marketing analytics, such as customer reviews (41, 42).

The topic classifier maps a text string, in our case the article title $T_{a}$, onto a normalized vector of topic likelihoods that span 25 different categories: Books, *CareerAndMoney*, *SocialMedia*, *FamilyAndFriends*, *Fashion*, *Fitness*, *FoodAndDrink*, *Health*, *Technology*, *Leisure*, *QuotesAndLifePhilosophy*, *Relationships*, *Movies*, *Music*, *PersonalMood*, *PetsAndAnimals*, *Politics*, *SchoolAndUniversity*, *SpecialOccasions*, *Sports*, *Television*, *Transport*, *Travel*, *VideoGames*, *Weather*. See the Mathematica “FacebookTopic” classifier’s description page for more details.

Roughly 14.5% of MC articles in our data sample featuring keywords ($e_{a}$) in the MC metadata—see Fig. S3e for the 100 most frequent entity tags. These granular entity tags support a cross-validation of the topic classifier. As a demonstration of consistency, Fig. S4 shows the correspondence between the classifier categories and the 10 most frequent $e_{a}$ tabulated across all articles classified according to a principal category. The correspondence is particularly strong in the case of the most frequent entity tag for each category. By way of example, for articles classified as having their principal category weight in the *Weather* (respectively, *PetsAndAnimals* and *Transport*) category, the most frequent $e_{a}$ is “weather” (respectively, “animals” and “roads and traffic”).

To reduce the effects of categorical overlap and improve visualization, we consolidated the estimated weights of the 25 categories into 10 refined topic categories, denoted by ${\vec{c}}_{a}$. To be specific, we merged the weights for categories *Travel*, *Transport*, *SpecialOccasions*, and *Leisure* categories into a single *Tourism* category; merged *Sports*, *Fitness*, *FoodAndDrink*, and *Health* into the *Recreation&Health* category; merged *Movies*, *VideoGames*, *Books*, *Television*, and *Music* into an *Media&Entertainment* category; merged *SchoolAndUniversity* and *CareerAndMoney* into *Education & Professional*; merged *Relationships*, *FamilyAndFriends*, and *Social Media* into a *Social* category; and merged *QuotesAndLifePhilosophy*, *Fashion*, and *PersonalMood* into a *Personal* category. Then for a given subset of articles, we combine the corresponding ${\vec{c}}_{a}$ to compute an average topic vector $\langle \vec{c}\rangle_{\;p}$ associated with that subset *p* (e.g. representing a given park in a given year).

### Testing the proportion of tourism-oriented topics between two complementary topic vectors

To test whether tourism-oriented topics are over- or under-represented when parks are co-visible, we calculate the average topic vector $\langle \vec{c}\rangle_{ij}$ from the $N_{ij}$ articles featuring parks *i* and *j*. We then denote $p_{1}=P_{ij,\text{Tourism}}$ as the topic vector weight corresponding to the *Tourism* category. Similarly, we also calculate an average topic vector across the $N_{i\cup j\setminus ij}=N_{i}+N_{\;j}-2N_{ij}$ articles corresponding to the complementary set $\left(\{N_{i}\}\cup \{N_{\;j}\})\setminus \{N_{ij}\right\}$ of articles featuring parks *i* or *j* but not both; from this vector, we also denote $p_{2}=P_{i\cup j\setminus ij,\text{Tourism}}$ as the topic vector weight corresponding to the *Tourism* category. To test for over-representation of tourism-oriented topics, we calculate the test statistic


(2) $$Z=\frac{\left(p_{1}-p_{2}\right)}{\sqrt{\;p_{1}(1-p_{1})/N_{ij}+p_{2}(1-p_{2})/N_{i\cup j\setminus ij}}}$$

and test the (one-sided) alternative hypothesis that Z>0 (i.e. $P_{ij,\text{Tourism}}>P_{i\cup j\setminus ij,\text{Tourism}}$), based on the assumption that difference in proportions is approximately normally distributed with mean $\left(p_{1}-p_{2}\right)$ and SD corresponding to the denominator of Eq. 2.

## Results

### Trends in visitation and federal budget support

The US NP system is a dynamic multiorganization that relies upon the effective allocation of personnel and other resources to reach its environmental and tourism objectives (20). Yet this fundamental task is complicated by the wide variation across the 63 distinct units, which extend over several orders of magnitude in size, traffic, park budget and public visibility—as illustrated in Figs. 1 (nominal values) and S5 (values calculated on a per-visitor basis). Such variability reduces the effectiveness of parsimonious resource allocation strategies—for example, whereas Gateway Arch (JEFF) in downtown St. Louis may be the smallest park by area at (0.9 km)^2^, it also ranks 15th in terms of visitation with upwards of 2 million visitors in 2019 alone.

Moreover, in addition to its immense scale, geographic fragmentation and structural diversity, the NP system is tethered to global tourist population growth and the increasing demand for tourism. Yet the persistent growth of ecotourism demand and public attention is countered by declining budgetary support—see Fig. 1b–g. To put this into perspective with numbers, the NPs witnessed a 29% increase in vehicle traffic over the last decade (2010–2019). In 2019, NPs welcomed twice as many tourists as New York City (>124 million visitors), distributed over an area >270 times as large—see Table S1. Yet after accounting for inflation, federal budget support decreased by 15% over the same decade. During the COVID-19 pandemic, as park visitation subsided while need for access to parks surged (43), the 2019 NPS current appropriations budget of 3.2 Billion $USD declined in 2020 by 14% (44).

### Structural representation of the US NP system

In order to generate a system-level perspective, we apply a digital media co-occurrence framework previously developed to analyze the contextual relationships between a collection of distinct entities, such as individuals (33) and universities (32). When extended across a system of entities, this method enables the use of digital media co-visibility as a quantitative and granular indicator of brand association, a key dimension of brand equity in marketing science (32, 45, 46). Consequently, by identifying park–park relationships through media articles that mention both parks, we can construct a coherent and dynamic representation of the system from the complete ensemble of park–park relationships as they evolve over time. As such, this approach leverages a foundational public-oriented data source to generate a structural representation of the US NP system, complementing a recent study analyzing the frequency of individual NPs featured in social media posts (47).

Our study is distinguished by its emphasis on the relational dimensions of NPs, particularly the degree to which tourism-oriented topics emerge when media coverage references two or more parks at the same time. To this end, we first examine the distribution of $N_{NP}$, the number of distinct parks co-occurring within a single article. We find that 86.7% of articles mention just one park, 8.9% mention two parks, and 4.4% mention three or more. Considered together, the frequency distribution $P(N_{\mathrm{NP}})$ follows a Zipf distribution—see Fig. S6a. Over time, the frequency of articles with $N_{\mathrm{NP}}>1$ has stabilized around 13%, despite the nominal rate of these multi-NP articles increasing exponentially, from roughly 300 per year in 2010 to over 10,000 in 2020. As such, in what follows we develop a dyadic NP–NP network that offers a valuable historical perspective on the structure and dynamics of NP system integration in the public sphere.

**Fig. 2.**
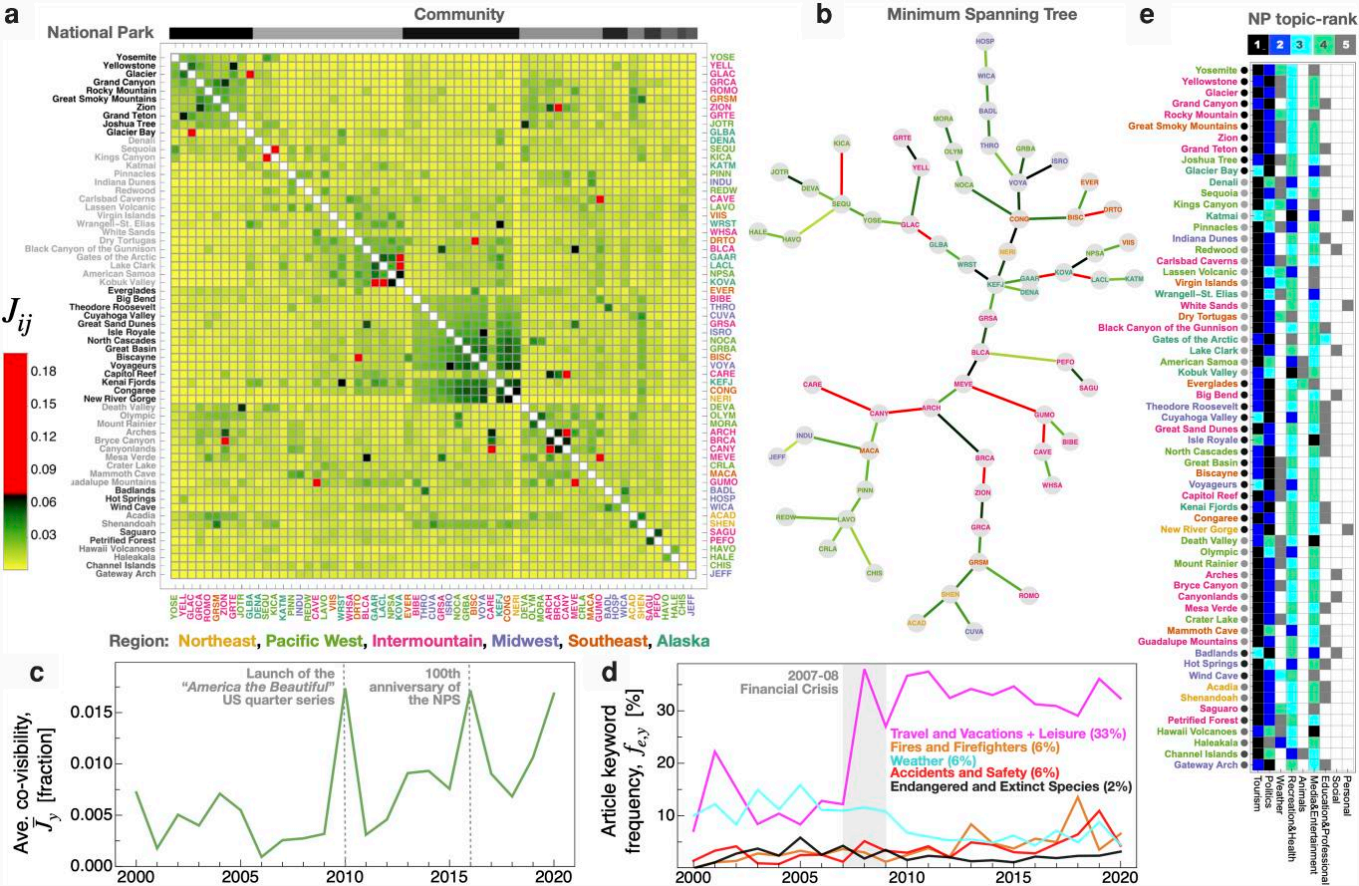
Structure and dynamics of NP system integration according to digital media co-visibility. a) The Jaccard similarity index $J_{ij}$ measures the fraction of media articles featuring parks *i* and *j* out of the total number of articles mentioning *i* or *j*. Shown is the full ensemble of $J_{ij,2010-2020}$ values calculated for articles published between 2010 and 2020. The strongest 10 links are highlighted at the extreme of the color scale to facilitate visual identification. Communities identified using the Louvain modularity maximizing algorithm (48) are indicated by the segments along the upper border; within each community, NP are sorted according to their total media visibility. Community members are largely correlated according to prominence and bio-geographic similarity and less by geographic proximity (NPS regions). See Fig. S9 for a dynamic visualization of Jaccard co-occurrence matrices at the 1-year resolution from 2000 to 2020. b) Minimum spanning tree illustrating the backbone of the $J_{ij,2010-2020}$ matrix, which is oriented around regional proximity; links are colored according to (a). c) The average value ${\bar{J}}_{y}$ quantifies the increasing integration of the NP system according to media co-visibility. d) The percentage of articles associated with fundamental aspects of the NPS mandate relating to visitation, recreation, and ecosystem management. The percentages shown in parenthesis capture the relative frequency of the corresponding keywords over the 21-year period. e) The NP-topic matrix exhibits the variation in NP media contextualization. Each row indicates the top-5 topic categories for a given park; circles on the left margin indicate the corresponding NP community shown in (a).

To examine the structural relationship between two distinct embeddings of this system (23)—namely, the spatial embedding based on regional proximity and the media co-visibility embedding reflecting the public imaginary—we applied an unsupervised, modularity-maximizing algorithm to detect communities of nodes characterized by dense internal connections relative to external ones (40, 48). Figure 2a shows the resulting community structure that emerges from the ensemble of $J_{ij}$ values, which is highlighted by four prominent communities featuring mixed geographic representation.

The ordering of parks within each community are sorted in decreasing order of $N_{i}$, such that the first park listed for each group (i.e. Yosemite, Denali, Everglades, Death Valley, etc.) is the park with the most prominent media visibility, independent of its co-visibility. Visual inspection of communities indicates stratification according to prestige and relatively weak correlation according to geographic proximity. In particular, the first community headed by Yosemite NP includes a number of “crown jewel” parks, such as Great Smoky Mountains NP, which attracted 23 million visitors in 2019—see Table S1.

A complementary view of the system structure emerges by examining the largest co-visibility values, which further elucidate the structural role of the most prominent NPs. To this end, Fig. 2b shows the minimum spanning tree (MST) representation of the $J_{ij}$ matrix generated via the Kruskal algorithm. This approach reveals the network backbone by retaining only the strongest connections while ensuring an acyclical structure (i.e. no loops) in the resulting sparse network representation (see (40) for details on this canonical network refinement method). Results indicate that many MST branches connect parks from the same administrative region, however many of the strongest $J_{ij}$ values do extend across the entire spatial embedding—see Figs. S7 and S8. Together, these patterns suggest two modes of system-level integration: prestige and proximity. With five of the six administrative regions containing parks from the “crown jewel” community, the most prestigious parks likely enhance the visibility of their regional neighbors. This suggests a brand association or “halo effect,” where increased attention to prestigious parks generates visibility and visitation spillovers for other proximal parks.

### Increasing pairwise and system-level integration

Whereas the spatial embedding of the NPs would suggests a fragmented and weakly interdependent system, the integration of the system within the public domain of digital media is substantial, which has implications for visitor dynamics and system management strategy. Supporting this perspective, a recent study demonstrated that higher NP visibility on social media (e.g. Twitter and Instagram) is linked to increased visitation (47). In a similar vein, our results indicate that NP co-visibility may capture unobserved patterns of co-visitation, potentially revealing tourist routes that extend both within and across regional boundaries.

While overall visitation levels are influenced by factors such as a park’s distance from metropolitan centers, economic climate, and the growth of international tourism (49), the latent structure and dynamics of $J_{ij}$ suggest three additional, less understood modes of multipark visitation. The first mode arises from the human desire to reproduce positive experiences, as the inspirational and shared experiences generated by visiting the most popular “crown jewels” organically promotes visitation to less prominent parks. The second mode manifests through explicit media messages recommending a group of parks based on shared features, such as exotic wildlife and trees, landscapes, and geological anomalies. And the third combines romantic ideals with the satisfaction generated through the process of collection and ownership. This tendency is captured by a 2016 Washington Post article which offers avid ecotourists a practical solution to the challenging “traveling salesman” problem that hinders multipark visitation, essentially serving as a treasure map for NP enthusiasts (50).

As such, the degree to which the NP system integrates has ramifications for visitor, budget, and brand management, as well as the development and efficacy of systemwide agenda-setting and administrative policy (13). As evidence of growing pairwise and system-level integration, the average value of the co-visibility matrix shown in Fig. 2c exhibits a substantial increase over time. For example, by 2020 the average co-visibility ${\bar{J}}_{2020}=0.017$ is roughly four times the average level of co-visibility from 2000 to 2009. Notably, ${\bar{J}}_{y}$ illustrates the sensitivity of NP visibility to marketing events, with 2010 being the inaugural year of the “America the Beautiful” US quarter coin series featuring a number of parks, and 2016 being the 100th anniversary of the NPS. Additionally, the annual sequence of $J_{ij,y}$ matrices provides a more complete perspective on the pace of structural integration both within and across administrative regions—see Fig. S9 for a dynamic visualization of the $J_{ij,y}$ matrix at the 1-year resolution from 2000 to 2020.

While increased integration reinforces NPS brand equity (32, 35), it also contributes to the tension faced by park administration charged with ecosystem preservation and risk management on the one hand, and visitor accessibility and recreational amenity services on the other. This paradox is highlighted in Fig. 2d, which shows the frequency of media articles associated with five park-management themes. Notably, media articles associated with tourism grew dramatically following the 2007–2008 financial crisis, which disrupted the news media industry, among others, giving rise to a diverse ecosystem of new media sources oriented around high-throughput content production and pay-per-click advertising (32, 51–53). In contrast, we do not observe similar media production increases associated with fire, climate, safety, and wildlife management that are central to the environmental conservation mandate of the NPS.

This key insight emerges from our systems-oriented analytical framework, since examining only the nominal frequency of park-management themes (i.e. article counts associated with endangered wildlife) could misleadingly suggest an overall increase in attention on this important theme, as total NP media production has risen dramatically over time—see Fig. 1f and g. However, when analyzed in relative terms across a comprehensive sample (i.e. as a percentage of all NP articles, as in Fig. 2d), our findings reveal a systematic crowding-out of news media promoting public awareness of the environmental conservation challenges, which has been substituted by a dominant messaging stream around tourism. Consequently, the growing prevalence of tourism-oriented messaging, likely designed primarily to stimulate visitation, may exacerbate the overall challenge of parks and protected areas management by reducing the efficacy of messages oriented around conservation initiatives and responsible park use, both of which are essential to public participation in parks and protected area stewardship.

### Which media topics contribute to NP system integration?

We applied topic modeling to the set of media article titles associated with each park to characterize its individual topical profile. Figure 2e highlights the five most prominent topics for each park, showcasing the wide variety of topic profiles across parks. One common trend is that *Tourism* or *Politics* often appear as the primary or secondary topic category. Whereas *Tourism* encompasses articles on travel accommodations, transportation and holiday vacations, the *Politics* category spans articles on government, institutional and legislative contexts. There is also notable variation in principal topics among members of the same community, indicating that co-visibility and narrative topics are distinct degrees of freedom.

**Fig. 3.**
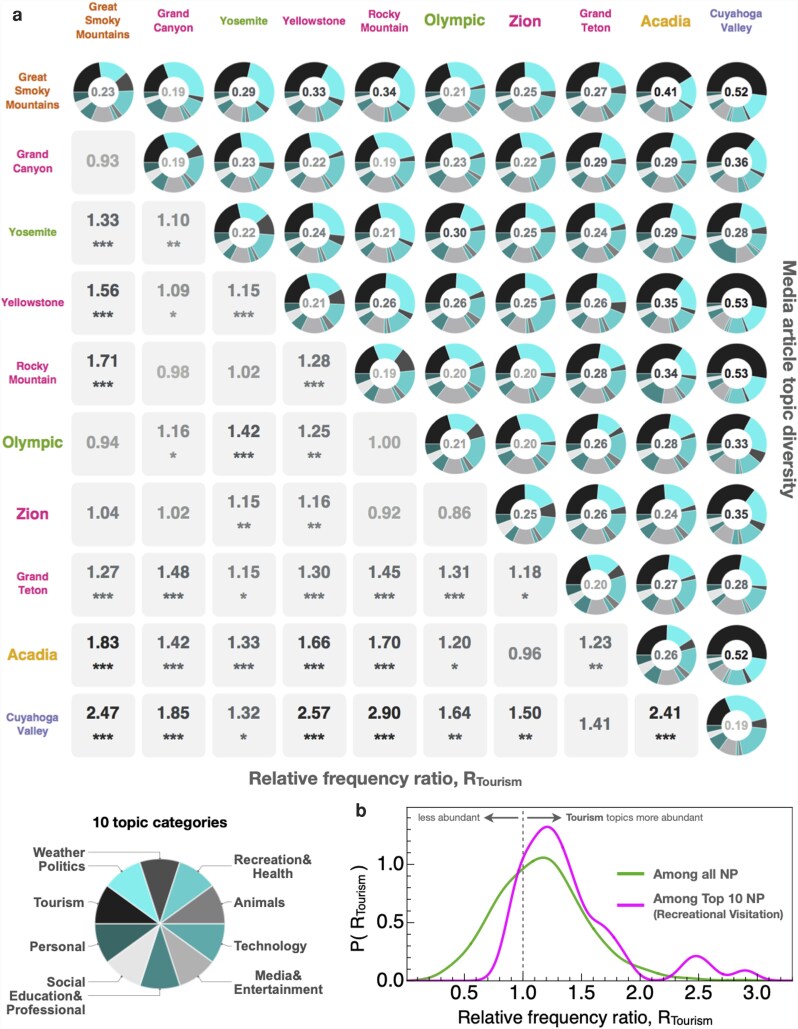
NP co-visibility promotes tourism in excess of other media topics. a) Topic-category frequency distribution for the 10 NPs with the highest levels of recreational tourism. The upper-diagonal (respectively, diagonal) elements of the NP–NP matrix feature a pie-chart showing the article title topic category distribution $P_{ij,c}$ calculated for the co-visible articles featuring parks *i* and *j* (resp., the distribution $P_{i,c}$ calculated for all articles featuring park *p*). At the center of each pie-chart is the frequency of the *Tourism* topic category, $P_{\;p,\text{Tourism}}$; the average and SD across the 63 parks is ${\bar{P}}_{\mathrm{Tourism}}=0.22\pm 0.05$. The corresponding lower-diagonal elements show the relative frequency ratio $R_{ij,\text{Tourism}}$, representing the degree to which *Tourism* articles are over-represented (R>1) or under-represented (R<1) by $P_{ij,\text{Tourism}}$ relative to the complementary frequencies $P_{i,\text{Tourism}}$ and $P_{\;j,\text{Tourism}}$. Stars indicate the statistical significance of R>1 values: ${\;}^{*}(P<0.05)$, ${\;}^{**}(P<0.01)$, and ${\;}^{***}(P<0.001)$. b) Distribution $P(R_{\mathrm{Tourism}})$ showing the frequency of $R_{\mathrm{Tourism}}$ values calculated among all NP pairs, as well as among the 10 NPs featuring the highest levels of recreational tourism shown in (a). Among all NP pairs, 25% have $R_{\mathrm{Tourism}}>1$ that are statistically significant at the P<0.05 level; among the top-10 NPs, this percentage increases to 76%.

Having defined the characteristic profile of each park, we then infer shared media narratives by analyzing the distribution of topics across the $N_{ij}$ articles linking each park pair. Figure 3a shows the 10 parks featuring the highest levels of recreational visitors. Along the diagonal of the graphical matrix, we show the distribution of topic vector weights averaged across all $N_{\;p}$ articles from 2010 to 2020 that feature park *p*, denoted by $\langle \vec{c}\rangle_{\;p}$. At the center of each pie-chart is the net share of *Tourism* topics, denoted by $P_{\;p,\text{Tourism}}$. Across all 63 parks, the minimum, maximum, and average ± SD value of $P_{\;p,\text{Tourism}}$ are: 0.14, 0.4, and 0.22±0.05, respectively—see Table S1. Similarly, the upper-diagonal elements of the matrix represent the average topic vector $\langle \vec{c}\rangle_{ij}$ calculated for the subset of $N_{ij}$ articles featuring both parks *i* and *j*, with the proportion of topics being tourism-oriented denoted by $P_{ij,\text{Tourism}}\in [0,1]$. Visual inspection indicates that articles featuring two or more parks are more likely to be tourism-oriented.

We test this pattern by comparing the set of co-occurring articles, denoted by $\left\{N_{ij}\right\}$, to the complementary set of articles, $\left(\{N_{i}\}\cup \{N_{\;j}\})\setminus \{N_{ij}\right\}$, according to the proportion of tourism-oriented topics they feature. As with the definition of $P_{ij,\text{Tourism}}$, we denote the proportion of topics being tourism-oriented in the complementary set as $P_{i\cup j\setminus ij,\text{Tourism}}$. We calculate the ratio of the two proportions,


(3) $$R_{ij,\text{Tourism}}=\frac{P_{ij,\text{Tourism}}}{P_{i\cup j\setminus ij,\text{Tourism}}}.$$

which are displayed in the lower-diagonal elements of Fig. 3a. Values with R>1 indicate a greater proportion of tourism topics in articles featuring both those parks relative to the complementary set. We then test the statistical significance of the R>1 values by way of a Z-test statistic calculated from the two proportions (see the Materials and methods section for more details).

Among the top 10 tourism-oriented parks, the average *R* value is 1.39, corresponding to a 39% excess of tourism-oriented topics in articles featuring both parks relative to the complementary baseline. In total, 34 of the 45 pairs (75.6%) feature R>1 with statistical significance at the P<0.05 level, which is reasonable given the selection of these 10 parks according to high levels of visitation. Yet when calculated across all 1,953 park pairs, the average *R* value is 1.16, with roughly 23.7% feature statistically significant R>1 values. While this second frequency is substantially lower than the frequency recorded among the parks characterized by the highest levels of tourism, it nevertheless corresponds to roughly a quarter of the co-visibility relationships in Fig. 2a that promote co-visitation as the dominant context.

**Fig. 4.**
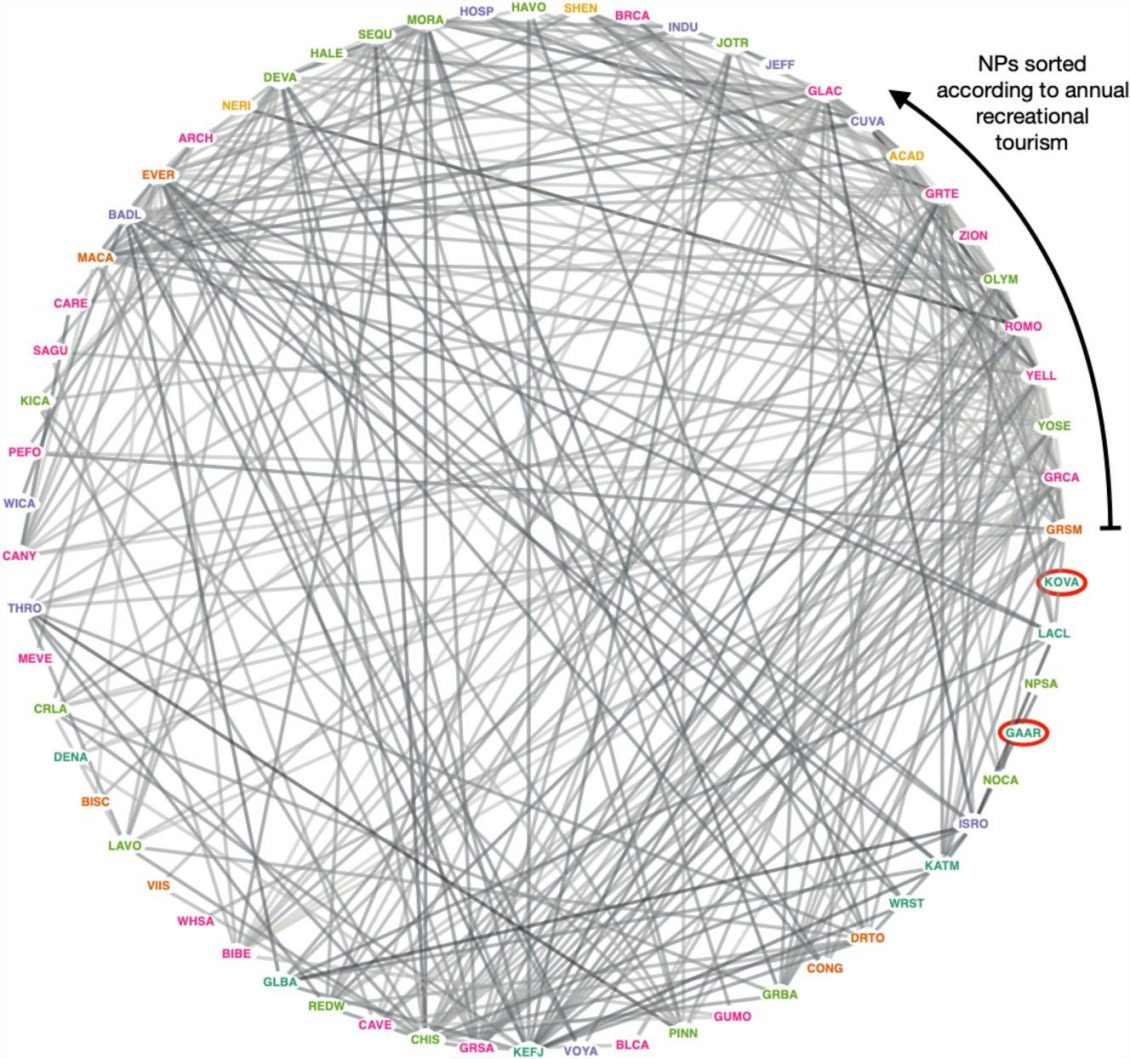
Network of NPs featuring excess co-visibility associated with “Tourism” media messaging. NPs are ordered counter-clockwise according to the total number of recreational visitors, starting with Great Smoky Mountains NP (GRSM). The presence of a link signifies that we calculate the relative frequency ratio $R_{ij,\text{Tourism}}>1$ at a statistical significance level of P<0.01. The large number of links spanning the center, as opposed to being constrained along the periphery, indicates a broad distribution of connectivity according to $R_{ij,\text{Tourism}}>1$ that is not simply explained by recreational tourism levels (i.e. the connectivity is dissassortative with respect to tourism intensity). Only two NPs (Kobuk Valley and Gates of the Arctic, which are circled) are disconnected from the remainder of the fully connected network. If we relax the significance constraint to P<0.05 level then these two NPs are also connected to the giant component.

Figure 4 shows the entire ensemble of park pairs featuring $R_{ij,\text{Tourism}}>1$ at the P<0.01 significance level. At this significance threshold, 61 of 63 parks are linked within a single connected component. Notably, connections span parks with varying levels of recreational tourism, indicating that tourism-oriented connectivity is dissassortative, a network characteristic that is attributed to resource constraints and zero-sum interactions (54), as documented in other multiorganizational systems (32, 39). Further relaxing the significance level to P<0.05 generates a fully connected network. This result suggests that tourism-oriented media messages—potentially associated with just a single park pair—could trigger visitation boosts that extend across the entire NP system.

## Discussion

The establishment of protected areas invests in natural capital by limiting resource extraction, land conversion activities, and other anthropogenic drivers of climate change (11, 55). With more than 17% of global land and inland waters having received protected area status (11, 27), this represents substantial progress towards limiting the degradation of global biodiversity and ecosystems deriving from human development (5, 16, 22, 56), and striving for targets set by 30×30 initiatives (25, 26). To this end, the 6,757 protected areas designated as NPs worldwide establish a vital interface between human-influenced and wild landscapes.

In addition to environmental conservation, protected areas also support societal and scientific objectives by maintaining cultural ecosystem services that provide present and future benefits to societal welfare (5, 57, 58). Considering the decline in both the number and diversity of nonprotected pristine biomes, the present and future value of protected areas is expected to increase (6), in particular when accounting for the value of ecosystem services as global expendable income per capita increases. Protected areas also generate ecological goods as *living laboratories* for conducting “wild science” (59, 60), thereby establishing invaluable biogeographical counterfactuals (11, 13, 59) that are essential to estimating the causal impact of anthropogenic pressures (e.g. climate change, invasive species, soil erosion, pollution) (13, 49, 61–68). This scientific value is reflected by recent shifts in NPS science policy that promotes a “Parks for Science, Science for Parks” synergy (61–64, 66, 68), thereby extending the 2-fold charge of the 1916 Organic Act into a 3-fold mission (13).

The US NPs span 1.6% of North America’s total land area and have shaped protected area management science by serving as an influential model for park systems worldwide (see Fig. S1). Yet NP systems are inherently complex to manage due to their multifaceted mandate, which defines the boundaries for responding to shifting environmental, social, and scientific agendas. Moreover, the heterogeneity of physical boundaries and visitor activities render individual parks difficult to protect (13, 21). These challenges compound as the number and separation of NPs increases within their overarching systems (22). Recent estimates indicate a density of one park ranger per 71.7 km^2^ in North American parks, which will need to increase to 1 ranger per 5 km^2^ to meet recommended standards given the skills they provide and role they serve as resource allocators (20, 69). For parks featuring relatively high levels of visitation growth, this target may be insufficient given that in addition to maintaining and safeguarding ecosystem integrity, biodiversity and physical infrastructure, park rangers are needed to protect tourists from natural risks and from themselves. And as social media has democratized content production, in particular in the outdoors adventure and recreation genre (47, 70–72), these and other public-generated data streams offer opportunities to better understand the coupled human-environment dimensions of NPs in order to address the tension generated by the conservation–recreation paradox facing the NPS.

In particular, there are scant resources for quantifying sociocultural relationships among parks, that together define the connectivity of the NP system. For example, a key yet underreported visitation metric is the number of different parks visited by a given NP tourist over a specific timeframe. Such data on NP entry and re-entry are necessary for optimizing the relative costs of an annual NP pass versus single entry prices and could identify the extent to which individual tourists connect the entire NP system. Insights could inform the development of more efficient budgeting and brand management by reconceptualizing NPs as administrative units that extend well beyond their geographic embedding (23).

To this end, in an effort to explore new data streams that can inform NP visitor management, we developed a framework to quantify the visibility and contextual narratives of protected areas in the public domain. A key result of our analysis is that media topics relevant to effective park management—concerning natural disasters, recreational safety, and the endangered wildlife that many parks specifically protect—have been overwhelmed by tourism media messages. On the aggregate, we find that 22% of media article topics across our sample are oriented around tourism—see Table S1 for $P_{\;p,\text{Tourism}}$ by park. While supporting tourism is central to the NPS dual mandate, achieving a more balanced media discourse that equally emphasizes conservation and stewardship is critical for fostering responsible and sustainable visitation. Future research could extend this approach through full-text analyses to assess how media narratives articulate principles of environmental stewardship consistent with sustainable ecotourism. Indeed, shifts in public communication modalities that amplify park visibility (47), and the recognition by NPS leadership to use social media as a tool to share and augment traditional news sources (49), have likely contributed to the 29% increase in NP visitation in the 2010s—see Fig. 1.

Our findings also highlight the potential impacts of increased NP co-visibility. With roughly 13% of media articles in our sample featuring two or more parks, this prevalence combined with the exponential growth of NP-associated digital media generates an effective marketing strategy tailored to systemwide tourism. This pattern is emphasized in mainstream media sources, which tend to feature larger numbers of parks within the same article—see Fig. S6. Consequently, our network analysis reveals that the NP system is highly interconnected through tourism-oriented media, implying that marketing efforts concentrated on even a single pair of parks could propagate visitation increases throughout the system. Together, these results suggest that rates of NP co-visitation within and across administrative boundaries are likely to increase over time.

## Conclusion

We analyzed a comprehensive corpus of digital media articles to construct a dynamic, systems-level representation of the US NPs. Using machine learning and network analysis, we identified latent connections among parks that extend beyond spatial proximity, thereby providing an integrated public-oriented lens for understanding the recreation–conservation paradox from a systems-level perspective. Our findings indicate that tourism-oriented media coverage has become a principal driver of park visibility and co-visibility in the digital age. However, as digital media visibility tends to focus attention on tourism-oriented topics, this risks diverting attention from critical conservation messages, such as environmental sustainability, wildfire management, and wildlife conservation. And while integration of parks within the public sphere may strengthen appreciation for parks and protected areas as a coupled human-environmental system, increasing media co-visibility may translate into rising co-visitation, amplifying visitation pressures on already constrained administrative capacities and fragile ecosystems amid declining budget support. These trends call for the NPS, alongside other federal agencies and park advocates, to ensure that promoting sustainable recreation becomes a central strategy for fulfilling its dual mandate, fostering a model of proactive public engagement in which visitors share greater responsibility for protecting the natural and cultural resources of the park system.

Given the rise of international ecotourism, our results can also inform both established and nascent NP systems developing globally (73), and whether they should embrace visitation as part of their mission, or just prioritize conservation. This consideration is critical for NP systems in under-resourced countries where infrastructure may not yet be in place to limit detrimental impacts of human activity.

From a policy and planning perspective, our framework underscores the need to expand visitor data infrastructure capable of capturing re-entry and co-visitation patterns, thereby overcoming the limitations of indirect system-level proxies such as digital media co-visibility. Such investments would enable a more holistic evaluation and administration of parks and protected area systems. In parallel, leveraging innovations in digital media through virtual visitation initiatives, such as digital tours and media engagement platforms (74), may offer sustainable pathways to manage visitor demand, broaden accessibility, and maintain public engagement. In doing so, these initiatives can complement broader efforts to promote sustainable recreation as a means of aligning visitor experience with long-term conservation goals. These strategies can help preserve the cultural and educational value of the parks while mitigating physical impacts and fostering a more equitable and resilient model of public stewardship. To effectively implement these measures, increased and sustained funding for data infrastructure, digital engagement, and adaptive visitor management programs will be essential to ensure that the National Park Service can meet rising visitation pressures while safeguarding environmental sustainability and public access.

## Supplementary Material

pgag028_Supplementary_Data

## Data Availability

All digital media article data are openly available from the Media Cloud project and are collected into a permanent Dryad digital repository, with DOI: 10.5061/dryad.fttdz091w; National Park visitation data are available via the NPS Integrated Resource Management Applications (IRMA) Portal. Mathematica code notebooks for reproducing figures are available in the permanent Dryad repository, with DOI: 10.5061/dryad.fttdz091w.
