# Supplementary material for: Increasing digital media visibility and tourism messaging promote US National Park system integration
Source: PNAS Nexus. 2026 Feb 10;5(2):pgag028. doi: 10.1093/pnasnexus/pgag028 (PMC12924136; doi:10.1093/pnasnexus/pgag028)
Supplement: pgag028_Supplementary_Data [file pgag028_supplementary_data.zip › PNASNEXUS-PNASNEXUS-2025-00556RR-s02.pdf]

Supplementary Information – Table S1 & Figures S1-S9

Increasing digital media visibility and tourism messaging promote US National Park system integration

Alexander M. Petersen[1,2], Felber Arroyave [1], Stephen Shackelton[2], and Jeffrey S. Jenkins[1,2]

[1]Department of Management of Complex Systems, Ernest and Julio Gallo Management Program, School of Engineering, University of California, Merced, California 95343, USA;

[2]National Parks Institute, University of California, Merced, California 95343, USA

U. S. National Park	NPS Abbrev.	Region	Year Founded	Total Visitors (10^6 persons) [2000-2020]	Visitors (10^6 persons) 2019	MC articles, N_p [2000-2020]	MM visibility, f_p^{MM30} [2000-2020]	$P_{p,\text{Tourism}}$ [2010-2020]
Yellowstone	YELL	Intermountain	1872	119.0	6.54	79588	0.11	0.21
Sequoia	SEQU	Pacific West	1890	26.77	1.56	9306	0.15	0.18
Yosemite	YOSE	Pacific West	1890	113.7	6.11	79843	0.14	0.22
Mount Rainier	MORA	Pacific West	1899	41.75	2.45	6938	0.19	0.18
Crater Lake	CRLA	Pacific West	1902	13.60	0.81	3074	0.15	0.22
Wind Cave	WICA	Midwest	1903	18.00	0.86	1340	0.12	0.18
Mesa Verde	MEVE	Intermountain	1906	12.61	0.65	3621	0.09	0.22
Glacier	GLAC	Intermountain	1910	53.13	3.46	37608	0.11	0.21
Rocky Mountain	ROMO	Intermountain	1915	76.87	4.90	22035	0.13	0.19
Haleakala	HALE	Pacific West	1916	25.87	1.03	1772	0.17	0.34
Hawaii Volcanoes	HAVO	Pacific West	1916	65.63	3.25	13318	0.09	0.17
Lassen Volcanic	LAVO	Pacific West	1916	10.71	0.62	2146	0.16	0.24
Denali	DENA	Alaska	1917	27.85	1.44	10548	0.12	0.31
Acadia	ACAD	Northeast	1919	58.83	3.69	9600	0.14	0.26
Grand Canyon	GRCA	Intermountain	1919	125.0	7.12	31290	0.15	0.19
Zion	ZION	Intermountain	1919	71.24	4.81	18788	0.14	0.25
Hot Springs	HOSP	Midwest	1921	74.52	3.72	4359	0.07	0.27
Bryce Canyon	BRCA	Intermountain	1928	42.75	3.12	5956	0.10	0.40
Grand Teton	GRTE	Intermountain	1929	99.76	5.58	17066	0.12	0.20
Carlsbad Caverns	CAVE	Intermountain	1930	8.83	0.44	2588	0.11	0.26
Everglades	EVER	Southeast	1934	22.43	1.16	12015	0.16	0.16
Great Smoky Mountains	GRSM	Southeast	1934	452.3	23.47	19579	0.11	0.23
Shenandoah	SHEN	Northeast	1935	33.12	1.74	5845	0.15	0.24
Olympic	OLYM	Pacific West	1938	90.65	4.37	9376	0.14	0.21
Isle Royale	ISRO	Midwest	1940	1.33	0.07	3491	0.10	0.14
Kings Canyon	KICA	Pacific West	1940	16.14	0.87	4474	0.16	0.19
Mammoth Cave	MACA	Southeast	1941	22.98	0.74	2223	0.13	0.23
Big Bend	BIBE	Intermountain	1944	10.72	0.63	6460	0.08	0.21
Virgin Islands	VIIS	Southeast	1956	17.69	0.37	1805	0.11	0.25
Petrified Forest	PEFO	Intermountain	1962	13.02	0.65	2572	0.18	0.23
Canyonlands	CANY	Intermountain	1964	12.37	0.83	3821	0.13	0.26
Guadalupe Mountains	GUMO	Intermountain	1966	4.12	0.21	1186	0.06	0.23
North Cascades	NOCA	Pacific West	1968	0.92	0.07	2481	0.16	0.20
Redwood	REDW	Pacific West	1968	8.98	0.51	2664	0.10	0.28
Arches	ARCH	Intermountain	1971	23.62	1.69	6794	0.13	0.28
Capitol Reef	CARE	Intermountain	1971	17.65	1.34	1733	0.14	0.33
Voyageurs	VOYA	Midwest	1971	5.64	0.28	1745	0.13	0.15
Badlands	BADL	Midwest	1978	21.35	1.06	4455	0.12	0.17
Theodore Roosevelt	THRO	Midwest	1978	12.43	0.74	6049	0.09	0.19
Biscayne	BISC	Southeast	1980	10.71	0.71	2084	0.15	0.19
Channel Islands	CHIS	Pacific West	1980	8.58	0.44	3500	0.10	0.21
Gates of the Arctic	GAAR	Alaska	1980	0.31	0.01	671	0.10	0.19
Glacier Bay	GLBA	Alaska	1980	9.53	0.69	2949	0.11	0.15
Katmai	KATM	Alaska	1980	1.27	0.09	3787	0.10	0.14
Kenai Fjords	KEFJ	Alaska	1980	5.81	0.36	1622	0.11	0.21
Kobuk Valley	KOVA	Alaska	1980	0.33	0.03	165	0.11	0.21
Lake Clark	LACL	Alaska	1980	0.27	0.02	667	0.11	0.23
Wrangell-St. Elias	WRST	Alaska	1980	1.26	0.07	1515	0.09	0.23
Great Basin	GRBA	Pacific West	1986	2.83	0.17	2174	0.12	0.21
American Samoa	NPSA	Pacific West	1988	0.28	0.06	490	0.10	0.22
Dry Tortugas	DRTO	Southeast	1992	1.57	0.09	1241	0.16	0.25
Death Valley	DEVA	Pacific West	1994	26.79	2.02	10925	0.12	0.17
Joshua Tree	JOTR	Pacific West	1994	40.95	3.33	15983	0.12	0.18
Saguaro	SAGU	Intermountain	1994	69.61	3.72	2874	0.17	0.27
Black Canyon of the Gunnison	BLCA	Intermountain	1999	4.93	0.46	833	0.13	0.24
Cuyahoga Valley	CUVA	Midwest	2000	54.13	2.25	4482	0.06	0.19
Congaree	CONG	Southeast	2003	2.53	0.16	1443	0.09	0.25
Great Sand Dunes	GRSA	Intermountain	2004	7.50	0.58	4430	0.07	0.18
Pinnacles	PINN	Pacific West	2013	4.27	0.18	3304	0.11	0.26
Gateway Arch	JEFF	Midwest	2018	52.92	2.06	1661	0.06	0.21
Indiana Dunes	INDU	Midwest	2019	41.08	2.16	2748	0.08	0.22
White Sands	WHSA	Intermountain	2019	10.41	0.61	1242	0.08	0.23
New River Gorge	NERI	Northeast	2020	24.38	1.20	805	0.10	0.17
**Summary**								
Average			1956	35.80	1.97	8,431	0.12	0.22
Standard Deviation			38	62.10	3.28	14,887	0.03	0.05
Total				2,256	124	531,147*		

TABLE S1. **U. S. National Park system**. List of 63 US National Parks in order of establishment year. Visitor data obtained from the NPS Integrated Resource Management Applications (IRMA) Portal. MC count indicates number of articles mentioning each NP by official name in the article's full text.
* Note that this tally is not the same as the number of unique articles (which is 426,069), as roughly 13% of the records feature 2 or more NP. The 8th column indicates the mainstream media prominence, f_p^{MM30}, which is the fraction of N_p published by 30 prominent national and regional news sources: "New York Times", "Wall Street Journal", "USATODAY", "Washington Post", "LA Times", "ABC News", "CBS News", "CNN", "FOX News", "MSNBC", "NBC News", "NPR", "Chicago Tribune", "Miami Herald", "San Francisco Chronicle", "Time", "The Boston Globe", "The Sacramento Bee", "The Seattle Times", "The Arizona Republic", "Huffington Post", "Salt Lake Tribune", "U.S. News", "The Fresno Bee", "The Denver Post", "Star Tribune", "San Jose Mercury News", "Washington Times", "The Desert Sun", "The Honolulu Advertiser".

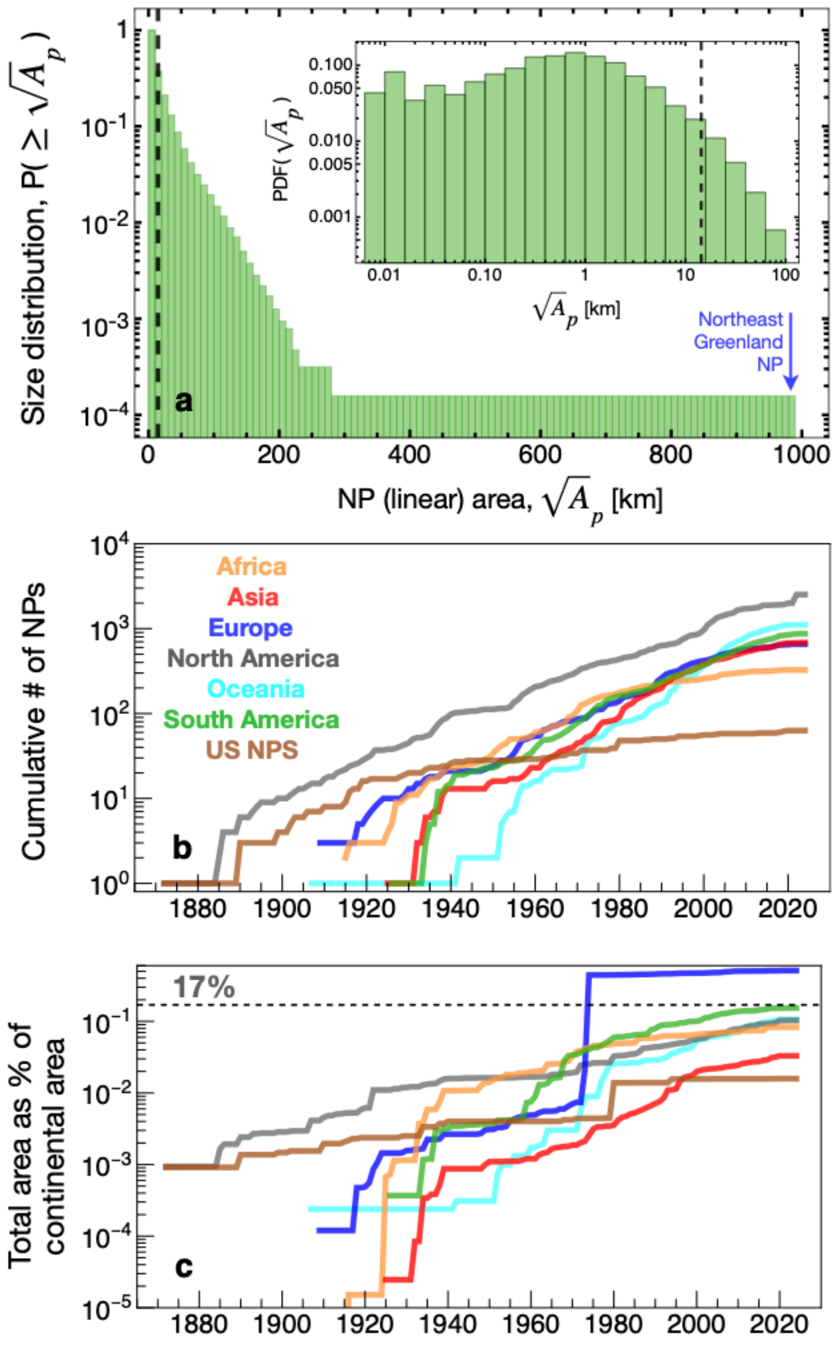

FIG. S1. **Global distribution and trends in the number and size of National Parks.** Prominent among the >287,000 non-marine PA units registered with the International Union for Conservation of Nature (IUCN) are the Category II territories distinguished as National Parks (NPs), which provide a valuable societal interface between human-influenced and wild lands. According to the 2024 IUCN registry, there are 6757 NPs formalized by 155 nations, representing 2.3% of all non-marine PA units but spanning 9.6% (6.4 million km^2) of the total global PA land area, thereby making substantial contributions towards the 17% PA objective set by Aichi Biodiversity Target 11 [9, 25]. **(a)** Size distribution of 6757 protected areas designated as NPs (WDPA Category II) using the most recent 2024 registry available from www.protectedplanet.net. To facilitate intuition, we report the size according to the linear distance ($\sqrt{A_p}$ in kilometers) associated with the reported total land area of each NP , which does not account for the complex geometry of the park boundaries [22]. Dashed vertical line indicates the mean value $\overline{\sqrt{A_p}}$ = 14.4 km, which is roughly the size of Mammoth Cave NP; the largest NP is (986 km)^2. (inset) The bulk of the size distribution follows a log-normal shape. **(b)** The total number of NPs per continent since the establishment of the first protected area via the *Yellowstone National Park Protection Act* of 1872. **(c)** Shown are cumulative areas normalized by the size of each continental area, demonstrating the degree to which NPs contribute to the 17% Aichi Biodiversity Target. US NPS refers to the subset of 63 NPs that together span 1.6% of the total area of North America. A notable recent addition in terms of its sheer size was the establishment of Wrangell-St. Elias NP in 1980, which at (184 km)^2 is roughly the area of Syria.

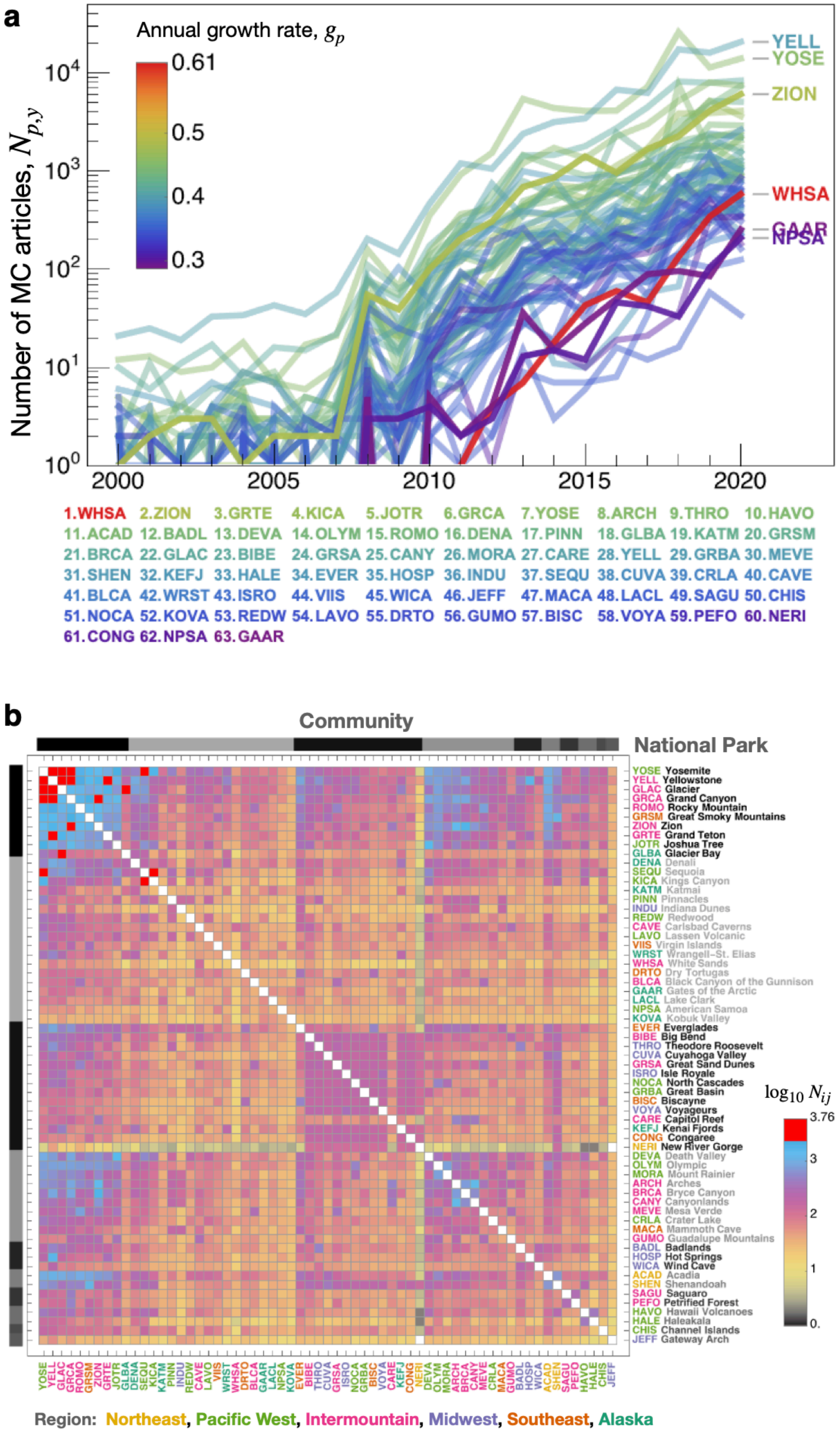

FIG. S2. **Digital media visibility and co-visibility of National Parks – nominal article counts.** **(a)** Number $N_{p,y}$ of MC articles mentioning each park p in year y. Each curve is colored according to its exponential growth rate g_p calculated at the 1-year resolution corresponding to the model $N_{p,y} \propto \exp[g_p y]$. NP are listed in rank-order of g_p to illustrate the size-growth variation. **(b)** The count matrix N_{ij} shows the number of media articles featuring NPs i and j, aggregated over 2010-2020. NPs are grouped according to the same clusters identified in **Fig. 2**(a), and are indicated by the gray-scale border segments along the upper border. Within each cluster, NPs are ordered according to their total media visibility, N_i. The top 10 N_{ij} values are indicated by red; the color index is shown in log scale, with the maximum value corresponding to 5,826 media articles associated with Yellowstone and Grand Teton.

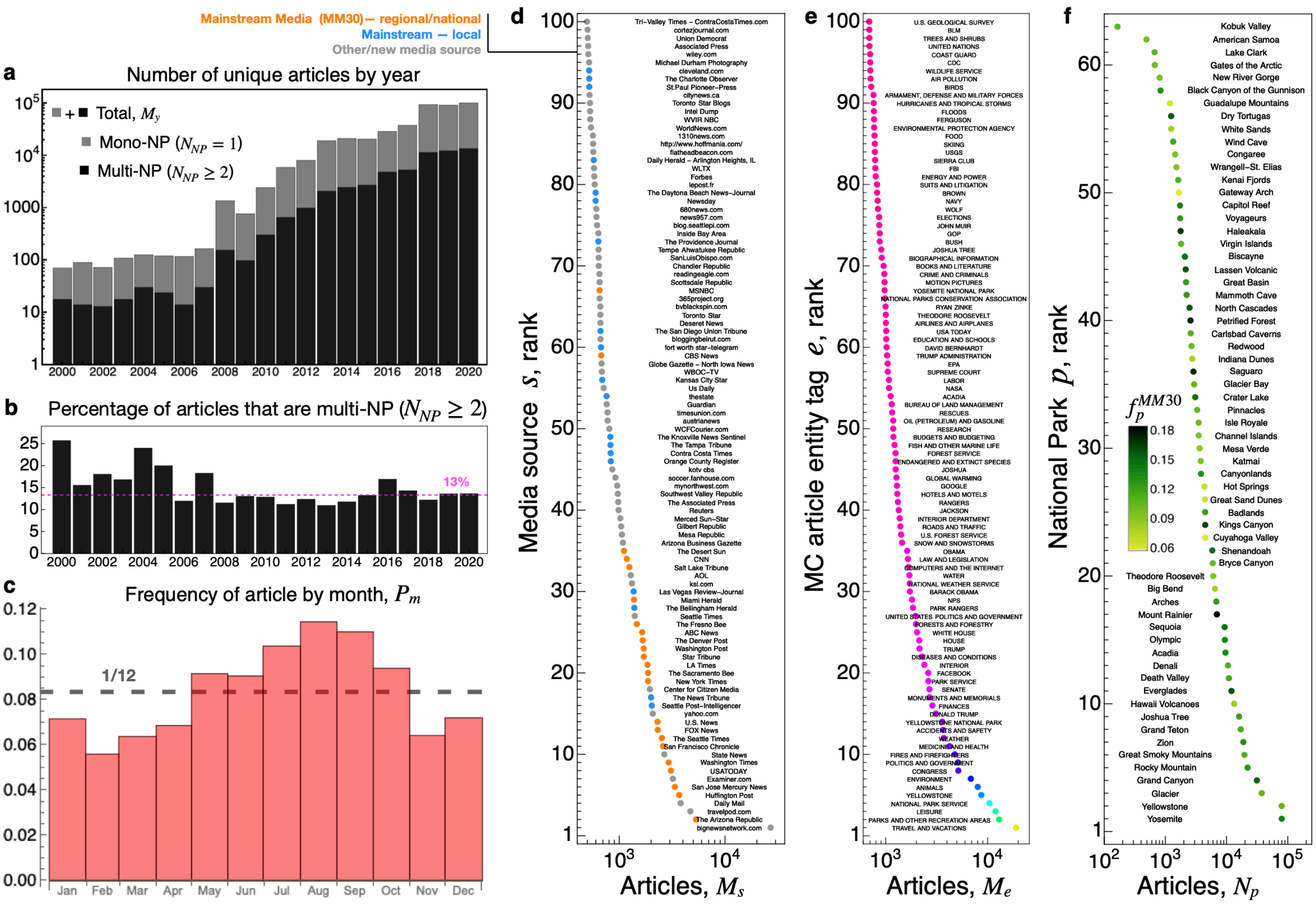

FIG. S3. **Covariates of national park media visibility.** Descriptive statistics including sample size by publication year and month, media source, and Media Cloud entity tags (analog to keywords). **(a)** The height of each bar indicates the total number of unique media articles by year, M_y. Complementary mono-NP and multi-NP (mentioning two or more parks) article subsets are indicated by the stacked bars heights. **(b)** Percentage of articles that are multi-NP ($N_{NP} \geq 2$); the dashed horizontal line represents the 13% average across all articles, corresponding to roughly 1 in 8 articles. **(c)** Frequency $f(m_a)$ of unique media articles by month. **(d)** The top-100 media sources by ranked according to the article count M_s. Colors indicate media source group: 30 select regional/national mainstream media sources ($MM30$, orange); local mainstream media (blue); other (gray). The $MM30$ group corresponds to 30 national and regional news sources manually selected according to their prominence and centrality within US states featuring large numbers of NPs; the Mainstream-local corresponds to the set of articles returned by MC using their internal media source filter for mainstream sources, less the MM30 group; and the “Other/new media source” group corresponds to the remaining media sources. To be specific, the $MM30$ group corresponds to: “New York Times”, “Wall Street Journal”, “USATODAY”, “Washington Post”, “LA Times”, “ABC News”, “CBS News”, “CNN”, “FOX News”, “MSNBC”, “NBC News”, “NPR”, “Chicago Tribune”, “Miami Herald”, “San Francisco Chronicle”, “Time”, “The Boston Globe”, “The Sacramento Bee”, “The Seattle Times”, “The Arizona Republic”, “Huffington Post”, “Salt Lake Tribune”, “U.S. News”, “The Fresno Bee”, “The Denver Post”, “Star Tribune”, “San Jose Mercury News”, “Washington Times”, “The Desert Sun”, “The Honolulu Advertiser”. **(e)** Media Cloud tags articles with keywords identifying distinct entities and overarching categories. Shown are the top-100 entities ranked according to article count, M_e. **(f)** NPs ranked according to the total number of media articles N_p featuring each park p over 2000-2020. Each data point is shaded according to the fraction f_p^{MM30} of the N_p articles that were published by MM30 sources.

Category (% of all articles)	Most frequent MC article tag (% of topic articles)	2	3	4	5	6	7	8	9	10
**Politics (21.)**	Donald Trump (20.)	Congress (17.)	environment (14.)	finances (12.)	animals (11.)	Senate (10.)	united states politics and government (9.4)	Interior (8.6)	law and legislation (7.5)	White House (7.5)
**Weather (8.9)**	weather (28.)	snow and snowstorms (18.)	National Weather Service (14.)	fires and firefighters (12.)	environment (7.5)	hurricanes and tropical storms (6.7)	floods (6.5)	animals (6.2)	forests and forestry (5.4)	accidents and safety (5.0)
**Travel (10.)**	accidents and safety (8.3)	diseases and conditions (7.8)	hotels and motels (7.2)	medicine and health (7.1)	airlines and airplanes (4.8)	Facebook (4.7)	animals (4.4)	fires and firefighters (4.0)	Donald Trump (3.8)	environment (3.3)
**Transport (6.2)**	roads and traffic (15.)	accidents and safety (14.)	animals (5.7)	environment (5.7)	weather (5.4)	fires and firefighters (4.6)	medicine and health (3.8)	Bureau of Land Management (3.8)	snow and snowstorms (3.3)	Facebook (2.9)
**PetsAndAnimals (3.0)**	animals (49.)	environment (13.)	medicine and health (10.)	fish and other marine life (9.3)	endangered and extinct species (8.4)	research (6.0)	accidents and safety (5.9)	birds (5.6)	rescues (5.1)	diseases and conditions (4.9)
**FoodAndDrink (4.1)**	environment (13.)	animals (11.)	Donald Trump (11.)	food (9.4)	medicine and health (8.7)	weather (7.4)	fires and firefighters (7.2)	restaurants (6.1)	water (5.0)	fish and other marine life (4.8)
**Health (3.3)**	medicine and health (36.)	diseases and conditions (27.)	animals (18.)	Felicia Fonseca (11.)	David Bernhardt (9.6)	Interior (9.5)	Lily Daniels (9.0)	CDC (8.3)	environment (8.1)	Donald Trump (7.6)
**Sports (3.5)**	animals (29.)	environment (11.)	endangered and extinct species (7.8)	accidents and safety (6.8)	Wildlife Service (6.6)	weather (5.0)	Park rangers (4.7)	insects (4.6)	NFL (4.4)	Office of the Federal Register (4.1)
**SchoolAndUniversity (2.7)**	education and schools (20.)	environment (11.)	Donald Trump (8.6)	medicine and health (7.8)	animals (7.2)	Congress (6.0)	research (5.6)	finances (5.2)	Betsy DeVos (5.1)	biographical information (5.0)
**Fitness (3.3)**	animals (13.)	El Capitan (7.8)	fires and firefighters (7.7)	Tommy Caldwell (7.0)	environment (6.8)	Kevin Jorgeson (6.4)	forests and forestry (5.3)	Congress (5.1)	accidents and safety (4.8)	Hans Florine (4.8)
**Movies (2.2)**	motion pictures (26.)	animals (17.)	environment (11.)	Alex Honnold (8.6)	Netflix (8.1)	fires and firefighters (7.0)	Tom Hanks (6.7)	Robert Redford (6.1)	recordings (audio) (6.0)	wolf (6.0)
**Relationships (1.2)**	computers and the internet (9.5)	crime and criminals (7.6)	murders and attempted murders (7.5)	medicine and health (7.3)	FBI (5.3)	google (5.3)	animals (5.2)	Congress (4.6)	fires and firefighters (4.6)	Alex Cox (4.5)
**CareerAndMoney (2.1)**	finances (23.)	Congress (14.)	Donald Trump (11.)	environment (9.3)	budgets and budgeting (9.0)	animals (7.9)	medicine and health (7.9)	Interior (7.0)	labor (6.9)	crime and criminals (5.3)
**Technology (1.9)**	computers and the internet (19.)	Google (11.)	animals (6.9)	telephones and telecommunications (6.2)	environment (5.9)	fires and firefighters (5.9)	computer software (5.4)	Congress (4.8)	medicine and health (4.3)	Apple (4.1)
**VideoGames (1.9)**	environment (16.)	fires and firefighters (11.)	animals (11.)	Donald Trump (8.5)	accidents and safety (6.7)	Congress (5.5)	finances (5.3)	budgets and budgeting (4.5)	park service (4.1)	water (4.1)
**Books (1.6)**	books and literature (18.)	Congress (9.3)	medicine and health (7.6)	animals (6.1)	Donald Trump (5.9)	environment (5.8)	Senate (5.0)	motion pictures (4.0)	murders and attempted murders (4.0)	Supreme Court (4.0)
**Television (1.4)**	fires and firefighters (14.)	animals (11.)	environment (8.6)	Donald Trump (7.9)	Redding Police (7.1)	Roger Moore (7.1)	television (6.3)	Thomas (6.1)	Chris Anthony (6.0)	James Sweeney (6.0)
**Music (0.97)**	music (27.)	recordings (audio) (11.)	animals (9.5)	biographical information (7.5)	reviews (7.5)	deaths (obituaries) (7.0)	rock music (6.8)	Dinah Washington (6.1)	Nat (6.1)	San Francisco Chronicle (6.1)
**FamilyAndFriends (1.2)**	children and youth (14.)	animals (12.)	Tylee Ryan (10.)	Chad Daybell (9.7)	Alex Cox (9.6)	fires and firefighters (8.7)	medicine and health (8.7)	Lori Vallow (8.3)	Kay Woodcock (7.2)	murders and attempted murders (7.2)
**SpecialOccasions (1.1)**	holidays and special occasions (13.)	Congress (9.7)	Paul Miller (8.4)	christmas (7.9)	Dawne Robinson (7.5)	Stephanie (7.2)	Joshua (6.8)	Joshua Tree (6.3)	environment (5.9)	trees and shrubs (5.8)
**SocialMedia (1.2)**	Donald Trump (9.3)	animals (7.4)	Congress (7.1)	fires and firefighters (6.4)	environment (5.7)	Facebook (5.4)	computers and the internet (5.3)	medicine and health (3.8)	Senate (3.5)	Joshua (3.3)
**QuotesAndLifePhilosophy (1.6)**	animals (17.)	environment (15.)	fires and firefighters (14.)	medicine and health (9.4)	forests and forestry (8.2)	Congress (8.1)	U.S. Forest Service (5.0)	weather (5.0)	trees and shrubs (4.7)	decisions and verdicts (4.6)
**Leisure (1.4)**	animals (12.)	environment (6.9)	fish and other marine life (6.2)	fishing, sport (5.7)	Facebook (5.1)	park rangers (4.9)	hotels and motels (4.4)	accidents and safety (4.3)	Disney (4.2)	holidays and special occasions (4.1)
**Fashion (0.57)**	animals (9.4)	medicine and health (8.0)	National Weather Service (7.7)	environment (7.4)	weather (7.4)	accidents and safety (7.1)	children and youth (7.1)	fires and firefighters (6.5)	electric light and power (6.2)	energy and power (6.2)
**PersonalMood (0.24)**	fires and firefighters (34.)	earthquakes (24.)	computers and the internet (20.)	forests and forestry (17.)	Arthur Gies (17.)	Bistra Dilkina (17.)	Detwiler (17.)	Ferguson (17.)	floods (17.)	housing (17.)

FIG. S4. **Classification validation.** Table showing 25 topic categories (listed in the first column), followed by the corresponding top-10 rank-ordered list of MC article-level entity tags (e_a). The values in parenthesis in the first column indicate the percentage of MC articles with principal classification belonging to a given category. Each row lists the top 10 e_a corresponding to that category, with percentage shown in parenthesis. By way of example, among articles classified principally by the *Weather* (respectively, *Fitness*) category, the second-most frequent e_a is "snow and snowstorms" ("El Capitan"), which occurred in 18% (7.8%) of those articles.

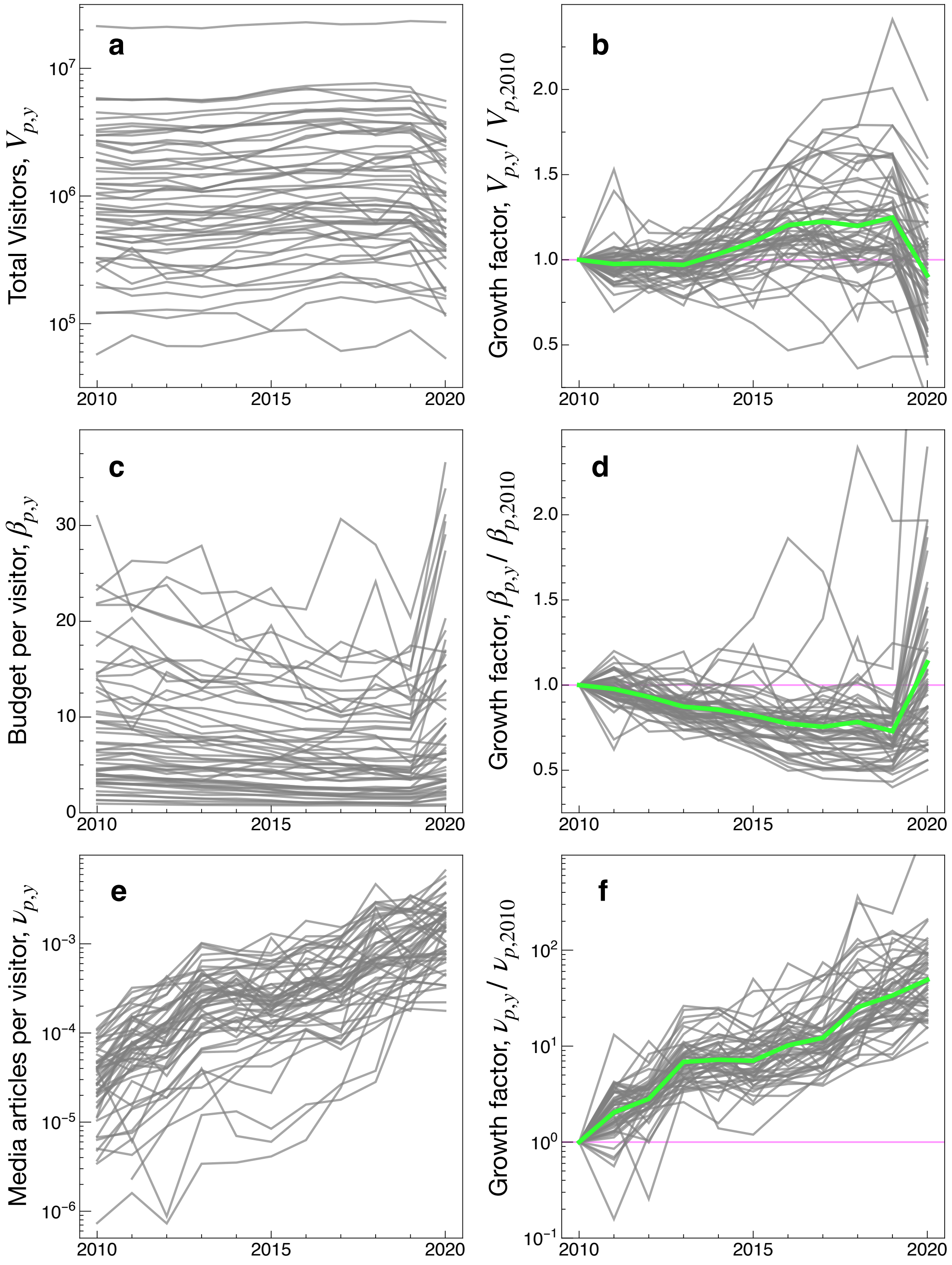

FIG. S5. **Trends in visitation (person headcount), federal budget per visitor, and media visibility per visitor.** **(a)** The total number of visitors (headcount) per park, $V_{p,y}$. **(b)** Visitor growth relative to 2010 levels. The solid green line represents the annual average: in 2019 the average increase in traffic was 25% above 2010 levels, $\overline{V}_{2019}/\overline{V}_{2010} = 1.25$; however, in 2020 visitor levels fell back to 91% of 2010 levels due to COVID-19 travel reduction combined with visitation permit systems implemented by the NPS, $\overline{V}_{2020}/\overline{V}_{2010} = 0.91$. **(c)** US federal budget per visitor, $\beta_{p,y} = B_{p,y}/V_{p,y}$. **(d)** Park budget growth relative to 2010 levels, $\beta_{p,y}/\beta_{p,2010}$. The average NP received 73% of its 2010 budget in 2019, but in 2020 this increased to $\overline{\beta}_{2020,y}/\overline{\beta}_{p,2010} = 1.13$ meaning that the relative magnitude of budget cuts were not as drastic as coinciding visitation declines. **(e)** The number of media articles per visitor, $\nu_{p,y}$. **(f)** Even when measuring park media visibility per capita, some parks witnessed nearly 2 orders of magnitude of growth relative to 2010. On average, parks received $\overline{\nu}_{2020}/\overline{\nu}_{2010} = 49$ times as much media visibility per visitor in 2020 as compared to 2010.

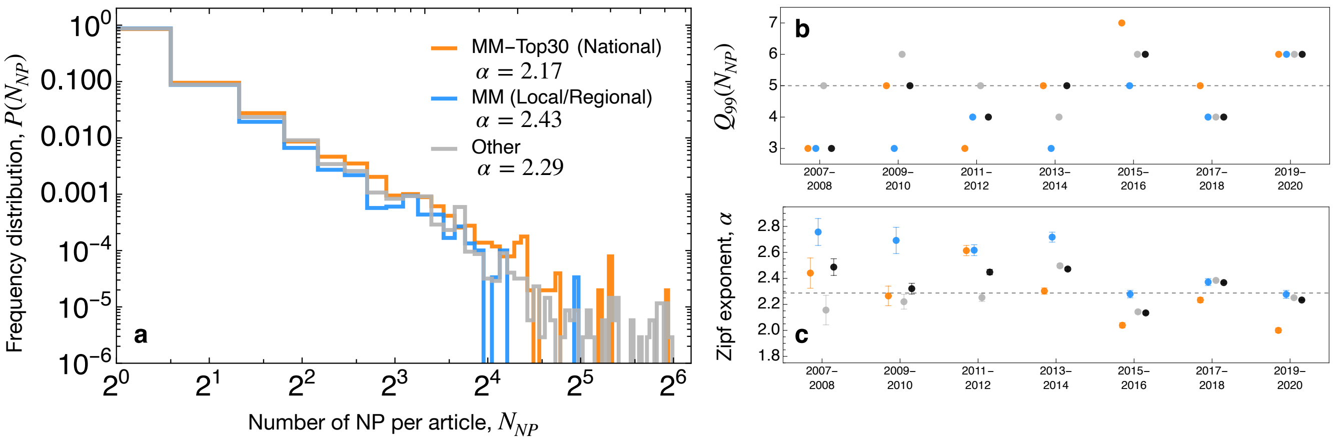

FIG. S6. **Frequency of multi-NP news media visibility.** **(a)** The average number of NP per media article is $N_{NP} = 1.25$. This single number does not readily convey the rich higher-order information contained in the full frequency distribution, $P_\alpha(N_{NP})$ – which is well-fit by the Zipf distribution $P_\alpha(N_{NP}) \sim 1/N_{NP}^{1+\alpha}$. This canonical Zipf count distribution is quantified by a single parameter, the scaling exponent α, which we estimate using the robust maximum likelihood estimator (MLE): the exponent calculated for the entire data sample is $\alpha_{\text{all}} = 2.29$. Larger α values signify a smaller likelihood of finding multi-NP news articles with $N_{NP} \geq 2$. In order to identify variation in $P_\alpha(N_{NP})$ according to the prominence and regionality of media sources, we disaggregated the full dataset according to three media source groups, denoted by g (see caption of Fig. S1 for their definition). Shown are empirical frequency distributions $P_\alpha(N_{NP}|g)$ conditioned on media source group g. Estimated scaling exponents α_g are indicated in the legend. **(b)** Evolution of the 99th percentile value $Q_{99}(N_{NP})$ calculated over 2-year non-overlapping periods from 2007 to 2020. The black dots correspond to $\alpha_{\text{all},y}$ calculated independent of g for a given period. **(c)** Evolution of α_g calculated over 2-year non-overlapping periods from 2007 to 2020. The black dots correspond to $\alpha_{\text{all},y}$ calculated independent of g for a given period. Error bars indicate the standard error in the MLE estimate. The aggregate trend for all media source groups combined (black points) is decreasing, which indicates that the frequency of multi-NP news is increasing over time. Comparing results across media source groups, the smallest value tends to occur for Top-30 MM media sources, meaning that their articles feature greater N_{NP}.

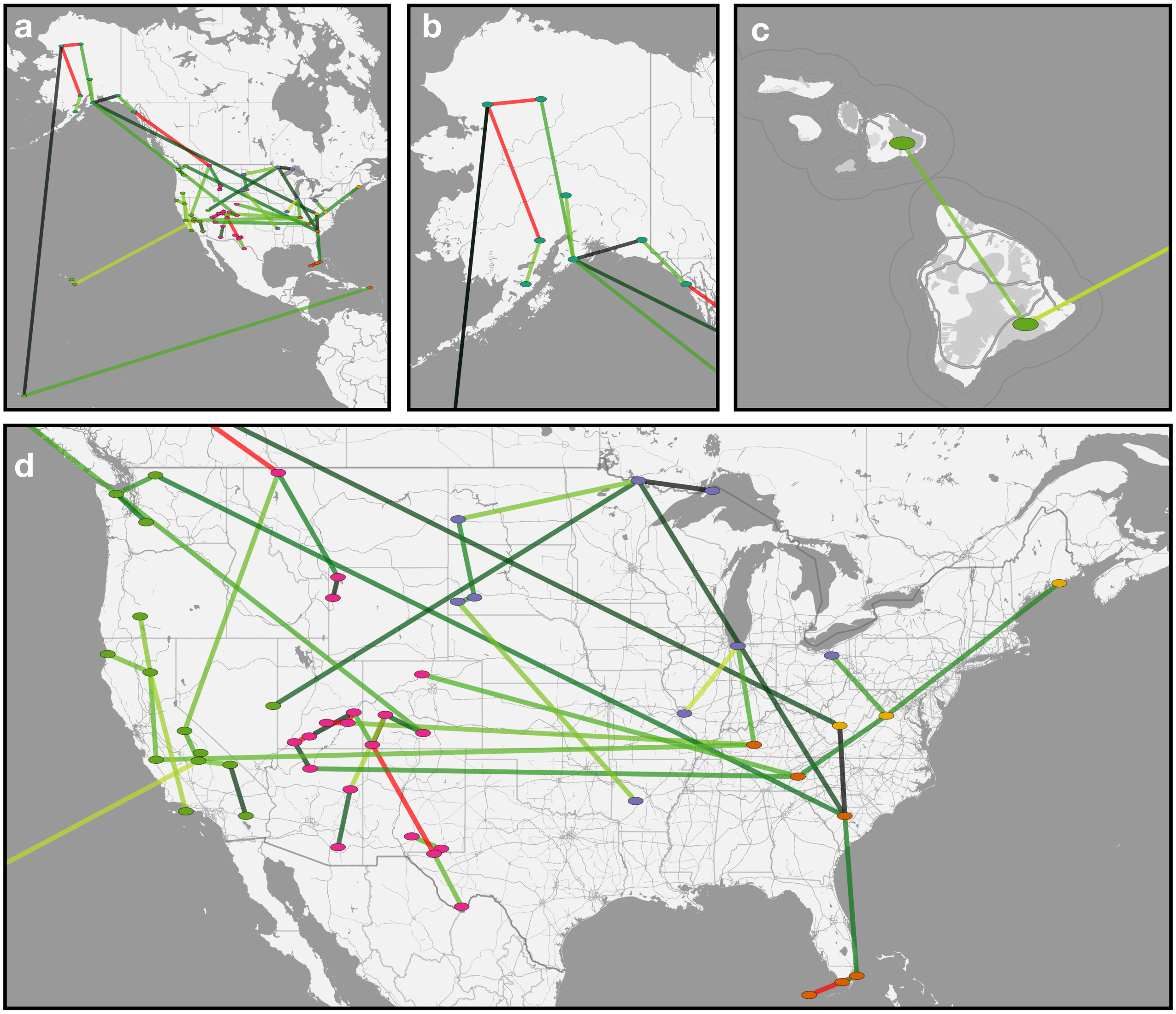

FIG. S7. **Spatial representation of NPS integration – MST. (a)** Geographic embedding of the minimum spanning tree (MST) shown in **Fig. 2**(b) illustrating the relational backbone of the NPS co-visibility network. **(b)** Alaska. **(c)** Hawaii. **(d)** Continental US.

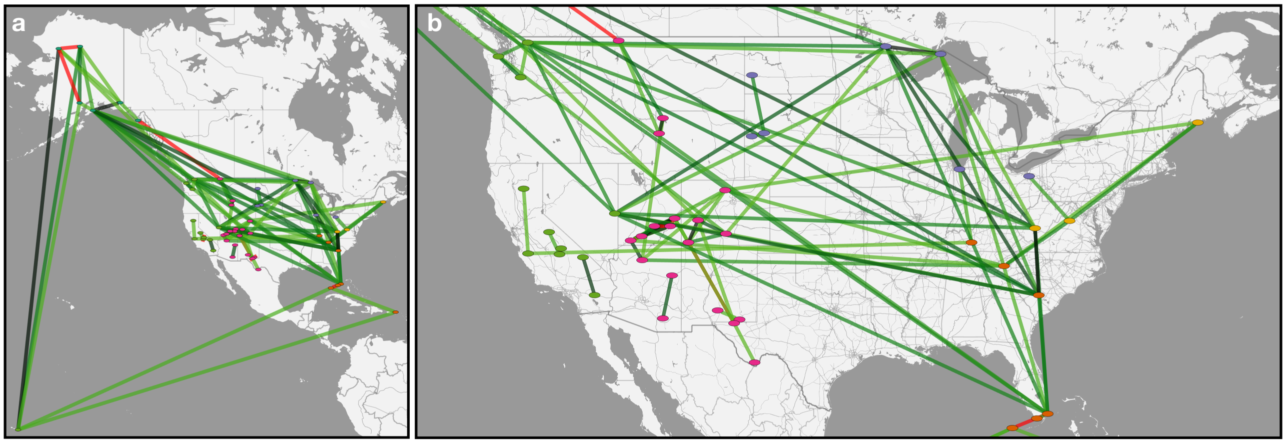

FIG. S8. **Spatial representation of NPS integration – top 5% strongest links. (a)** Spatial network constructed from the strongest NPS co-visibility network links – spanning just 54 parks. **(b)** Continental US.

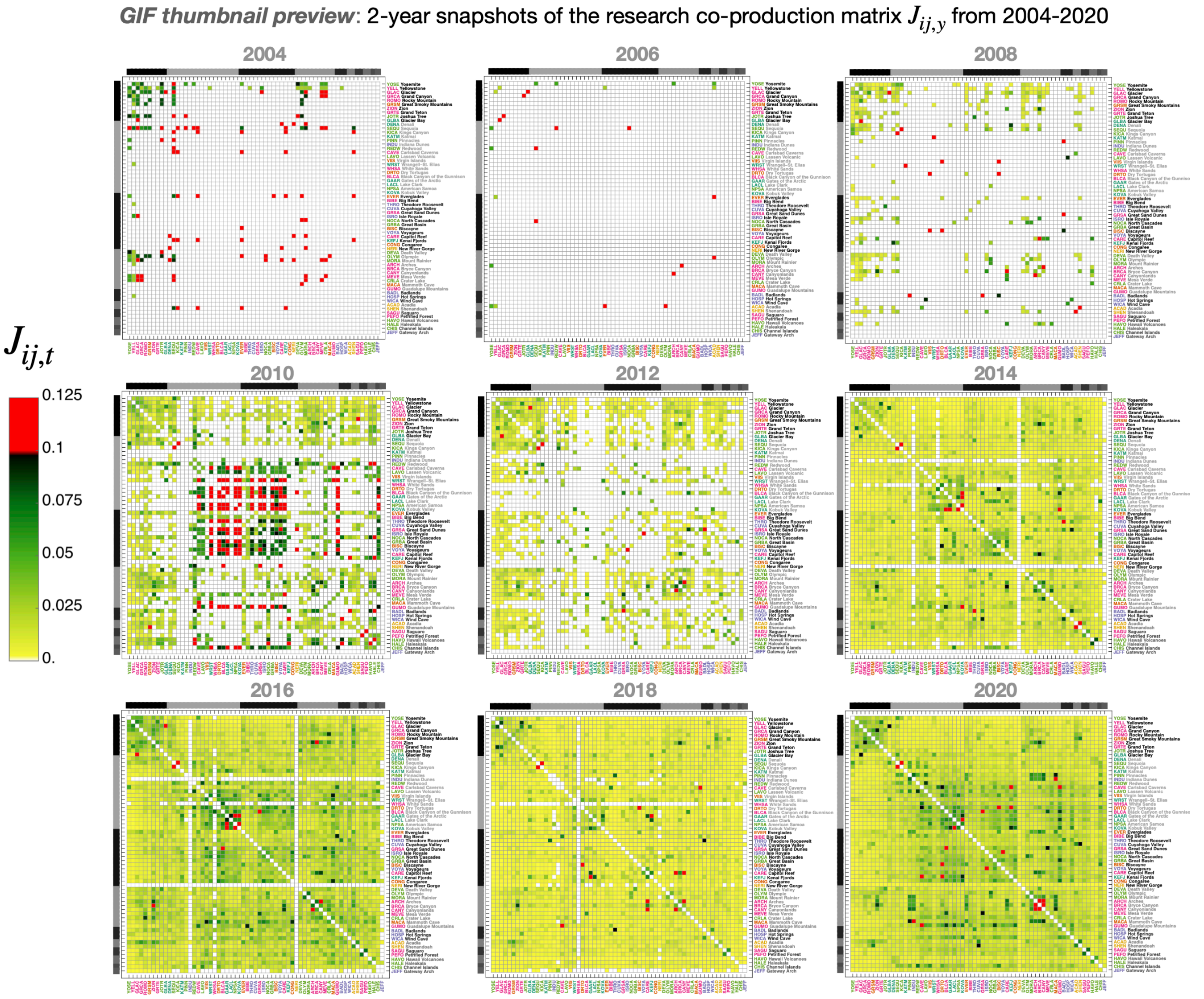

FIG. S9. **Dynamics of co-visibility matrix** $J_{ij,y}$. Shown are 2-year interval snapshots from the GIF image sequence contained in the supplementary file *JaccardMatrix_Y_2000-2020_Annual.gif*, which shows $J_{ij,y}$ calculated at the 1-year resolution from 2000 to 2020 with fixed order. We apply a two-regime color scale to facilitate identifying the most prominent NP pairs in a given year, such that red values indicating $J_{ij,y} > 0.1$, meaning that more than 10% of the media articles mentioning one or the other NP mentioned them together.
