# Supplementary figures and images for: Increasing digital media visibility and tourism messaging promote US National Park system integration
Source: PNAS Nexus. 2026 Feb 10;5(2):pgag028. doi: 10.1093/pnasnexus/pgag028 (PMC12924136; doi:10.1093/pnasnexus/pgag028)

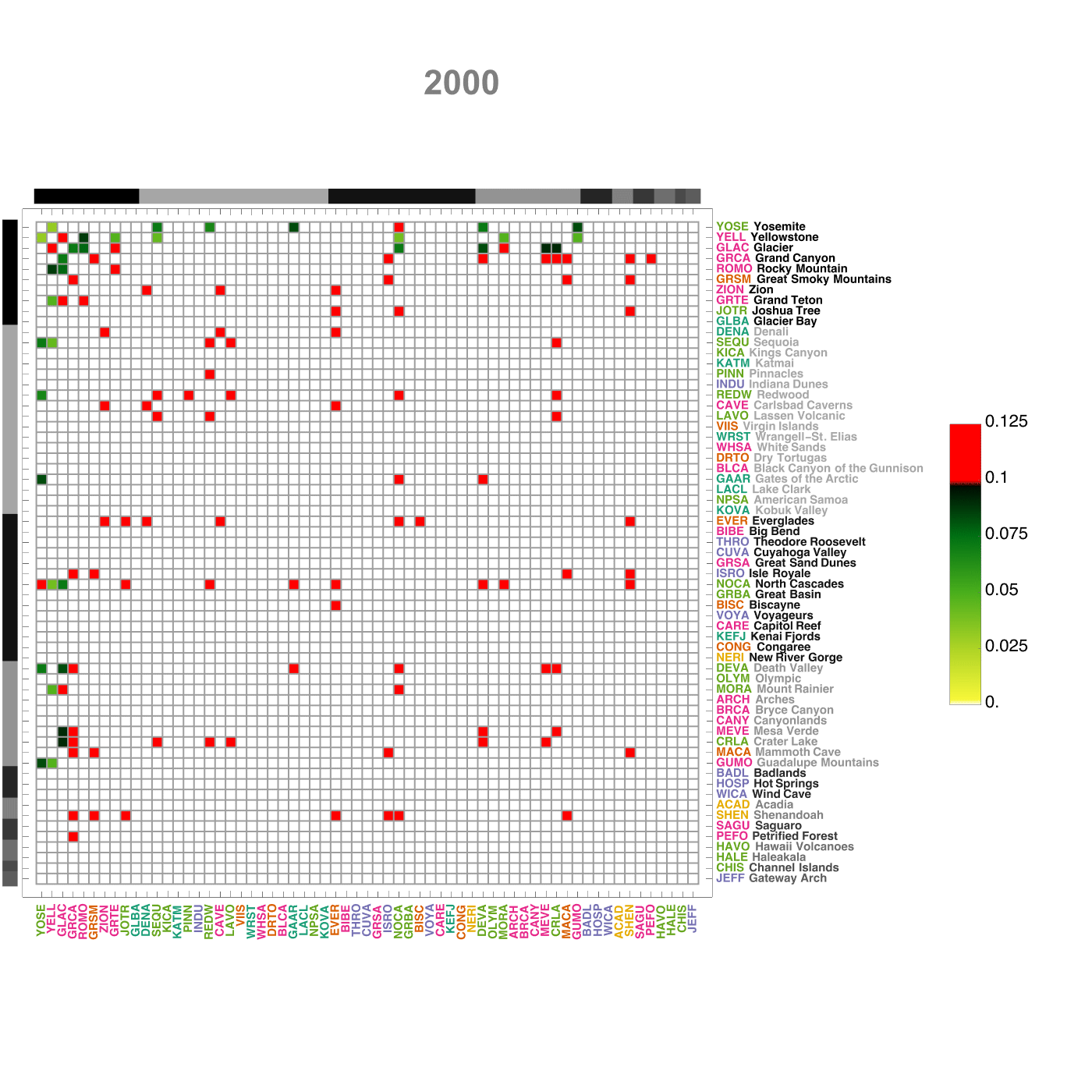

Supplement: pgag028_Supplementary_Data [file pgag028_supplementary_data.zip › PNASNEXUS-PNASNEXUS-2025-00556RR-s01.gif]
